# Single-cell characterization of CRISPR-modified transcript isoforms with nanopore sequencing

**DOI:** 10.1186/s13059-021-02554-1

**Published:** 2021-12-06

**Authors:** Heon Seok Kim, Susan M. Grimes, Anna C. Hooker, Billy T. Lau, Hanlee P. Ji

**Affiliations:** Division of Oncology, Department of Medicine, Stanford University School of Medicine, CCSR 1115, 269 Campus Drive, Stanford, CA-94305 USA

**Keywords:** Single-cell long-read CRISPR screen, Single-cell CRISPR screen, Single-cell long-read sequencing, Single-cell sequencing, Alternative splicing, Transcript isoform

## Abstract

We developed a single-cell approach to detect CRISPR-modified mRNA transcript structures. This method assesses how genetic variants at splicing sites and splicing factors contribute to alternative mRNA isoforms. We determine how alternative splicing is regulated by editing target exon-intron segments or splicing factors by CRISPR-Cas9 and their consequences on transcriptome profile. Our method combines long-read sequencing to characterize the transcript structure and short-read sequencing to match the single-cell gene expression profiles and gRNA sequence and therefore provides targeted genomic edits and transcript isoform structure detection at single-cell resolution.

**Supplementary Information:**

The online version contains supplementary material available at 10.1186/s13059-021-02554-1.

## Background

For any gene’s messenger RNA (mRNA), alternative splicing events lead to a diverse set of transcripts that have different combinations of exons. Also referred to as transcript isoforms, these different mRNA species result from genetic variants at intron-exon junctions and the functional contribution of different splicing factors. This diversity of transcripts provides cells with an added level of transcriptional regulation. Frequently, different isoforms have significantly different functions depending on the cell and tissue type. To date, most studies examining the structure and function of isoform variation have relied on CRISPR or other genome engineering methods. These experiments involve introducing a single genetic variant within an exon/intron junction or knocking out a splicing factor. Subsequently, the impact on transcript structure is assessed using conventional short-read RNA-seq—this bulk sequencing method averages the changes in transcript expression across many cells [1, 2]. Importantly, conventional short-read approaches may not resolve important transcript isoform features that are present in only a subset of cells since these require complex bioinformatic methods to resolve. Thus, it remains a challenge to determine how these factors influence the generation of different mRNA species.

Single-cell RNA sequencing (scRNA-seq) has been used for profiling transcript isoforms across individual cells [3, 4]. Some studies have adapted long-read sequencing for single-cell transcript isoform analysis [5, 6]. These approaches rely on instruments such as the Oxford Nanopore or Pacific Biosciences sequencers that generate long reads, typically over hundreds of bases if not much longer. Intact cDNAs are generated from single cells and used for library preparation without the fragmentation step required for short-read analysis. Sequencing the intact cDNA across the length of a single read can identify transcript structure and expressed genetic variants. However, this method only assesses the transcripts, without providing a way to experimentally test possible isoform alterations such as exon-intron junction modifications or splicing factor knock-outs.

Several studies have integrated single-cell RNA-seq with CRISPR screening to evaluate the transcriptional phenotypes of cellular networks [7–10]. These single-cell CRISPR studies require sequencing an individual cell’s expressed guide RNA; this barcode functions as an indirect indicator of which cells have a CRISPR edit. However, these methods do not directly verify the genome edit for a given cell. In addition, those approaches rely on short-read sequencing of transcripts where only a small portion of the ends of the mRNA are available. As a result, short-read sequencing methods for single-cell analysis do not identify changes in transcript structure nor the quantitative expression of individual isoforms.

**Fig. 1:**
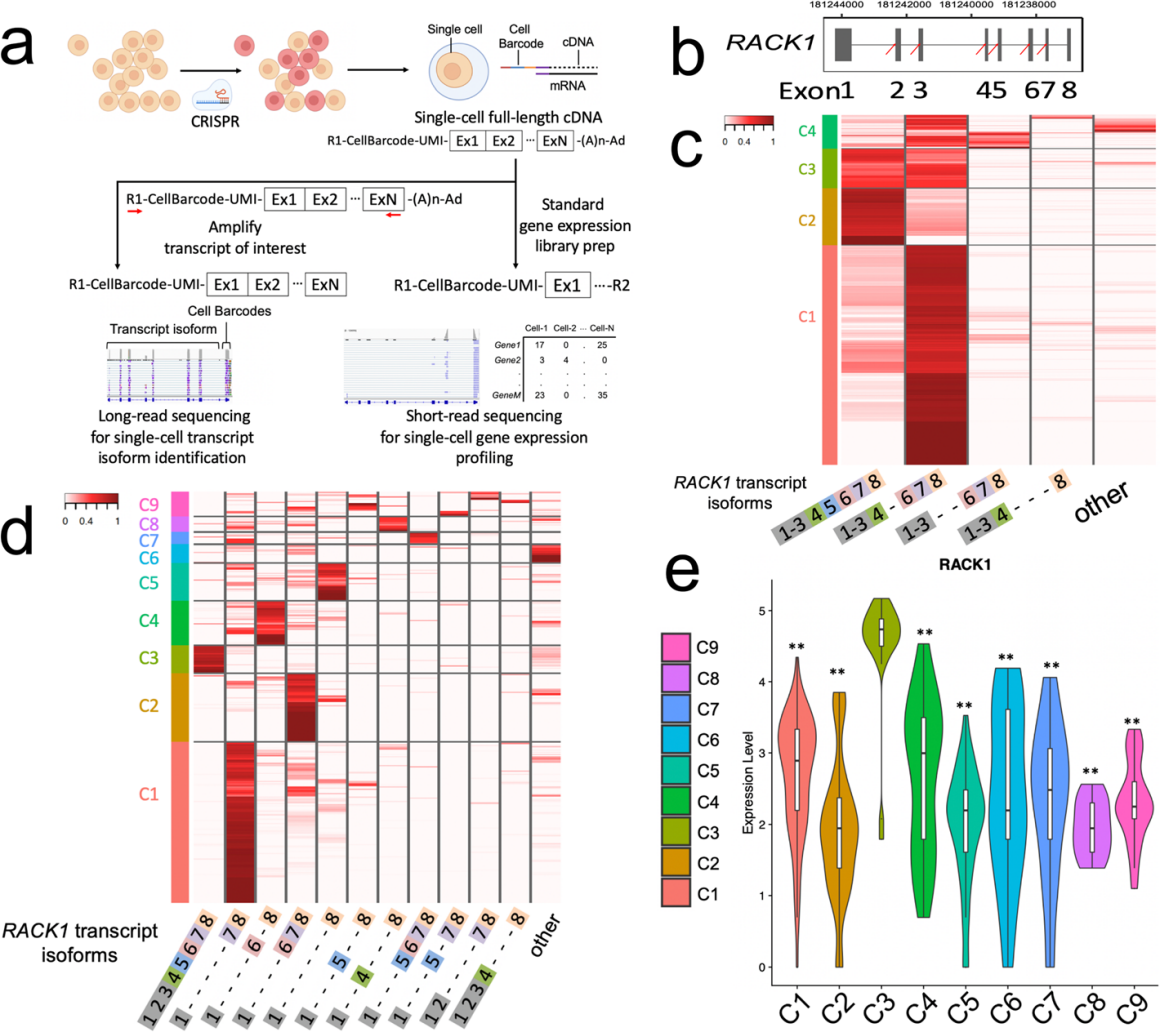
**a** Overview of single-cell short/long-read integration strategy of *RACK1* targeting. **b** Structure of *RACK1* transcript. **c, d** Two heatmaps showing the proportion of each transcript isoform (*x*-axis) with each cell (*y*-axis) for either the single (**c**) or multiplexed (**d**) *CRISPR* edits respectively. For panel **c**, hierarchical clustering was conducted on the cells subject to a single CRISPR edit of exon 5. Cluster 2 has the intact *RACK1* isoform with all exons. Cluster 1 cells demonstrate exon 5 skipping. For panel **d**, hierarchical clustering was conducted on cells subject to multiplexed edits across different exons. Cluster 3 indicates the cells with an intact *RACK1* transcript. The remaining clusters demonstrate cells with exon skipping. **e** Expression level of *RACK1* in each cell cluster denoted in panel **d**. *P* values were calculated for each cluster in comparison with Cluster 3 (C1 *P* = 5.7e−12, C2 2.6e−11, C4 9.9e−11, C5 1.6e−10, C6 1.1e−07, C7 9.5e−06, C8 2.5e−07, C9 1.1e−08 ; two-sided *t*-test)

Herein, we present a new approach which characterizes CRISPR-induced alterations of full-length transcript isoforms from single cells. We design CRISPR edits targeting specific exon-intron junctions that potentially alter transcript structure. As another experimental manipulation of transcript isoforms, we use CRISPR to alter specific splicing factor genes and assess how these changes influence a downstream gene’s expression of transcript isoforms. Our method relies on single-cell nanopore sequencing where an individual read has sufficient length to cover the entire mRNA molecule. We use this added information to extrapolate the transcript structure. Subsequently, we match each individual cell’s long-read information with its corresponding short-read transcriptome information to identify general transcriptional profile changes (Fig. 1A). Likewise, we show that CRISPR edits introducing short insertions or deletions (indels) are detected within sequences distal from the cDNA’s 5′ or 3′ end that could not be detected with short reads. This allows for validation of CRISPR edits even if they do not affect isoform sequence.

## Results

### Altering *RACK1* isoform structure with CRISPR and single-cell level detection

As a proof-of-concept, we designed and conducted an experiment targeting the splicing acceptor of exon 5 within the *RACK1* gene (Fig. 1B and Additional file 1: Fig S1). This gene encodes a receptor for activated C kinase 1. It plays a role in intracellular protein shuffling and anchoring. This gene was chosen because it is one the most highly expressed genes in the human embryonic kidney 293 T (HEK293T) cell line as determined from single-cell short-read gene expression data. In addition, it has eight exons to target with CRISPR. We selected a sequence for this target and generated a plasmid construct expressing Cas9 and target gRNA. These plasmids were transfected into HEK293T cells. After 6 days, we prepared single-cell cDNAs from both the wild-type and transfected cells with the gRNAs (Methods). From the single-cell cDNAs, the *RACK1* transcript was amplified using two primers that included sequence from the 5′ adaptor and the last exon of *RACK1*. The amplified target cDNA underwent long-read sequencing (Oxford Nanopore). We performed base calling using the program guppy and aligned the long reads to the reference genome, GrCh38 using minimap2 [11, 12].

Utilizing the cell barcodes at the 5′ end of the cDNA enables assignment of a long read covering a transcript to its cell of origin. Since these barcodes will not align to the genome, they should fall into the soft-clipped region of the aligned read. Accordingly, the soft-clipped portion of each read was extracted and compared to the whitelist of filtered cell barcodes identified by cellranger (Methods) from the short-read single-cell sequencing. The comparison was performed using vectorization with token length of 8 and a cosine similarity metric as described for assessment of text document similarity and referred to as tf-idf cosine [13]. The cosine similarity score evaluates the frequency of 8-mer occurrence between the whitelisted barcodes and the soft-clipped sequence, with higher scores indicating higher correlation. The edit distance between each of the top five scoring barcodes and each 16-bp region of the soft-clipped sequence was calculated and the barcode having the minimum edit distance was determined. If this best barcode had an edit distance less than three, it was considered a valid match; otherwise, the read was discarded from further processing.

Size of the *RACK1* amplicon from HEK293T cells aggregated in the distribution with mean 1126 and 5% and 95% quantiles of sizes length population are 1044 and 1212 respectively, which is the expected range. The mean edit distance of selected barcodes was 0.51 (Additional file 1: Fig S2). The cell barcode’s position within the soft-clipped portion of the read was an average of 0.54 bases from the expected position based on the known single-cell adapter/barcode construct. Overall, the data integration between single-cell long and short-read data enabled us to assess those cells with an altered target isoform and their accompanying full transcriptome features.

Using long-read sequencing of target cDNAs, we characterized the transcript isoform diversity in each individual cell. Prior to this quantification step, the 10 bp UMI sequence immediately following each cell barcode was reviewed. For any given set of reads that had the exact same barcode and UMI, these duplicate sequences were discarded (Methods). To assess the extent of potential UMI sequencing errors, the edit distance between UMIs in cells was calculated and plotted (Additional file 1: Fig S3). The edit distance approximated a random distribution with only a few UMIs per cell having edit distance less than 3 between them. Reads per cell barcode and their associated isoforms were then aggregated, and hierarchical clustering was used to determine the distribution of different isoforms among cell subpopulations.

Among the wild-type cells, we determined that nearly all of the cells expressed the full-length *RACK1* isoform. Specifically, 5030 out of 5074 cells had more than 90% of isoforms with full-length *RACK1* transcripts. However, for a small subset (Cluster 2) of wild-type cells, 44 out of 5074 cells demonstrated transcript isoform heterogeneity, averaging 23% of isoforms skipping one or more exons (Additional file 1: Fig S4). This result demonstrated how long-read analysis combined with scRNA-seq characterizes the underlying transcript isoform diversity present within individual cells.

For the cells transfected with CRISPR targeting the splicing acceptor of *RACK1*’s exon 5, 6028 of the 7548 cells had more than 33% of their *RACK1* isoforms lacking exon 5. This result showed how long-read analysis directly confirmed the presence of the genome edit (Fig. 1C). We performed hierarchical clustering based on the expression levels of the different *RACK1* transcript isoforms present in each cell. A subset of cells (Cluster 1, 4732 out of 7548 cells) contained CRISPR edits in the splicing acceptor in all three alleles of *RACK1.* A subpopulation of Cluster 2 cells (1234 out of 7548 cells) had predominantly full-length transcripts (82% on average), indicating a lack of CRISPR editing. One factor contributing to this subpopulation not being completely homogeneous may be the introduction of ambient RNA from the highly expressed *RACK1* gene being incorporated into the cell droplets and causing some cross-contamination.

### Detection of transcriptional changes by *RACK1* isoform perturbation

We studied the downstream transcriptional changes for the CRISPR-transfected cells with different *RACK1* isoforms. For this analysis, we integrated the long-read sequencing of *RACK1* cDNA with short-read sequencing of the matching single-cell transcriptomes. This step involved matching cell barcodes between the two data sets as previously described. Next, we compared *RACK1* expression level among the clusters of cells. The cells lacking edits (Cluster 2) had a 1.53-fold higher level of *RACK1* expression (*P* = 1.04e−194) compared to the cells in other clusters with edits (Fig. 1C and Additional file 1: Fig S5). The lower RACK1 expression among CRISPR-edited cells was likely the result of mis-splicing and nonsense mediated decay (NMD). When we directly compared gene expression profile of cells with the *RACK1* isoform lacking exon5 (Cluster 1) versus the full-length transcript (Cluster 2), we identified 177 differentially expressed genes including *RACK1* (Additional file 5: Table S4 and Additional file 1: Fig S6).

### Altering *RACK1* isoform structure with multiplexed CRISPR and single-cell level detection

Given this successful proof-of-concept, we designed a multiplexed CRISPR assay that targeted different exons in *RACK1*. We selected gRNAs that target the sequence of splicing acceptor sites of *RACK1* exons 2-7. We then transfected a pooled plasmid library containing these gRNAs into HEK293T cells with stable Cas9 expression (Methods). As previously described, single-cell cDNA libraries were generated from these CRISPR-transduced cells. Then, we used long-read sequencing of *RACK1* cDNA to determine isoform representation across individual cells. Our results indicated that the multiplexed CRISPR gRNAs generated edits leading to different exon-skipping events (Fig. 1D). The cells in Cluster 3 predominantly expressed full-length *RACK1* transcripts (83% full-length transcripts per cell on average), thus indicating that this subpopulation had not undergone genome editing. Other cells had reads indicating multiplexed CRISPR editing of splicing acceptors. For example, cells in Cluster 1 showed evidence of genome editing in exons 2 to 6 with the disruption of multiple splicing acceptor sites or large deletions (83% with an exon 2 to 6 skipped transcript per cell on average).

Then, we compared short-read gene expression profiles among the cluster of cells defined by the different *RACK1* isoforms (Fig. 1D). Using the short-read data, we compared the *RACK1* expression levels between the non-edited cells versus edited cells. As expected, *RACK1* expression among the non-edited cells (Cluster 3) was significantly higher than CRISPR-edited cells (6.59 fold change, *P* = 1.85e−9) (Fig. 1E). These experiments demonstrated that our approach allows us to identify CRISPR edits leading to new transcript isoforms as well as their transcriptional level at single-cell resolution.

### Single-cell CRISPR screen for various splicing factors with long-read sequencing

Different splicing factor genes impact the generation of alternative splicing events. We leveraged this aspect of isoform regulation to demonstrate another application of our approach. Namely, we determined how different splicing factors affect alternative splicing events of a downstream target gene. This mechanism of isoform generation is particularly crucial for immune cells where changing the regulator state and increased functional flexibility is paramount. For this experiment, we chose to study the *PTPRC* gene. Expressed in T cells, PTPRC is a transmembrane phosphatase and its pre-mRNA alternative splicing is critical for changing T cell regulatory states [14]. *PTPRC* has five highly expressed isoforms (Additional file 1: Fig S8). This includes two shorter ones where there is substantial degree of exon loss and several longer isoforms where the majority of exons from the variable region are retained. Naïve T cells preferentially express isoforms including variable exons like RB. On the other hand, activated or memory T cells undergo more exon skipping and express RO as a major isoform [14].

To study how various splicing factors impact the generation of *PTPRC* isoforms, we selected a series of gRNAs targeting a set of 16 splicing factor genes (Additional file 6: Table S5). We chose splicing factors which are expressed and play a critical role pre-mRNA processing in T cells [1, 15, 16]. For example, *CELF2* and *RBFOX2* regulate various pre-mRNA processing and other 14 splicing factors are known to interact with *PTPRC* pre-mRNA. We integrated this multiplexed CRISPR assay with single-cell long-read sequencing and determined how each splicing factor contributed to changes in *PTPRC*’s transcript isoform structure. For these experiments, we used the Jurkat human cell lines derived from a T cell leukemia. This line stably expressed Cas9. We transduced a multiplexed gRNA lentiviral library targeting 16 splicing factor genes (two gRNAs per gene) and five non-targeting gRNAs as negative controls (Additional file 6: Table S5). After 14 days, we harvested the cells, generated single-cell libraries, and conducted sequencing with both short and targeted long reads of *PTPRC*.

### Direct single-cell detection of CRISPR-induced indel mutations with long-read sequencing

**Fig. 2:**
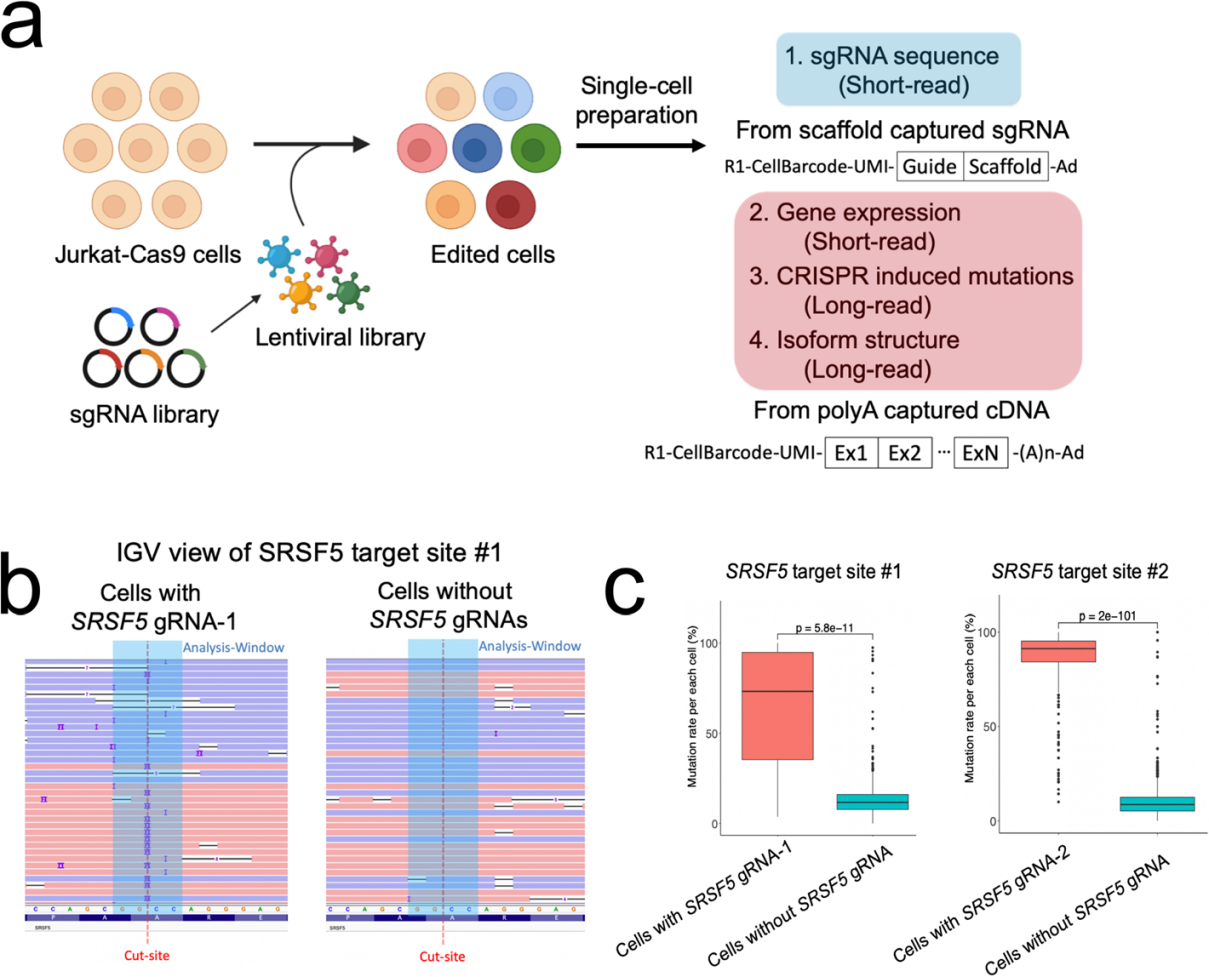
**a** Overview of single-cell CRISPR screen integrated with long-read sequencing. **b** IGV view of *SRSF5*-1 target site for cells containing different gRNA sequences. Reads from cells with *SRSF5*-1 gRNA show CRISPR-induced insertion (purple) and deletion (black line). CRISPR cut site and analysis window for statistical analysis are shown. **c** The percentage of mutated reads per each cell are shown as a box plot

First, we determined which gRNAs were expressed within a given individual cell (Fig. 2A). This method relies on using a primer to polymerase extend over the gRNA adjacent to a given cell barcode and then sequencing the product to obtain both in a given read (Additional file 7: Table S6) [17]. With the paired gRNA and cell barcode sequence, one determines the distribution of expressed gRNAs across individual cells. To assess the reliability of our long-read cell barcode matching process, we amplified our 16 targeted genes, conducted nanopore sequencing, and then identified the CRISPR-induced mutations at the gRNA target site (Additional file 1: Fig S9). In parallel, we conducted the single-cell short-read sequencing, determined the gRNA for each cell, and matched the short with the long-read cell barcodes. Based on this comparative analysis, CRISPR mutations were introduced at the target site at a significantly increased rate in cells with the appropriate target gRNA versus cells without the guide (Additional file 8: Table S7).

For example, we confirmed that cells expressing the *SRSF5*-1 gRNA had a variety of indels at the targeted Cas9 cleavage site in the transcript. This result contrasted to cells with other gRNAs which lacked indels in the target (Fig. 2B). Our quantitative analysis showed that the average mutation rate per cell with *SRSF5*-1 gRNA was 64.32% compared to 12.73% of cells with gRNAs targeting other genes at *SRSF5*-1 target site (Fig. 2C). The difference in mutation frequency was evident and highly significant (*P* = 5.85e−11) despite the high rate of error in nanopore sequencing. Overall, this result indicated that targeted long-read sequencing identified the CRISPR genotypes and that the short-/long-read barcode matching was reliable.

### Direct detection of CRISPR-induced *PTPRC* transcript isoform perturbation using single-cell long-read sequencing

**Fig. 3:**
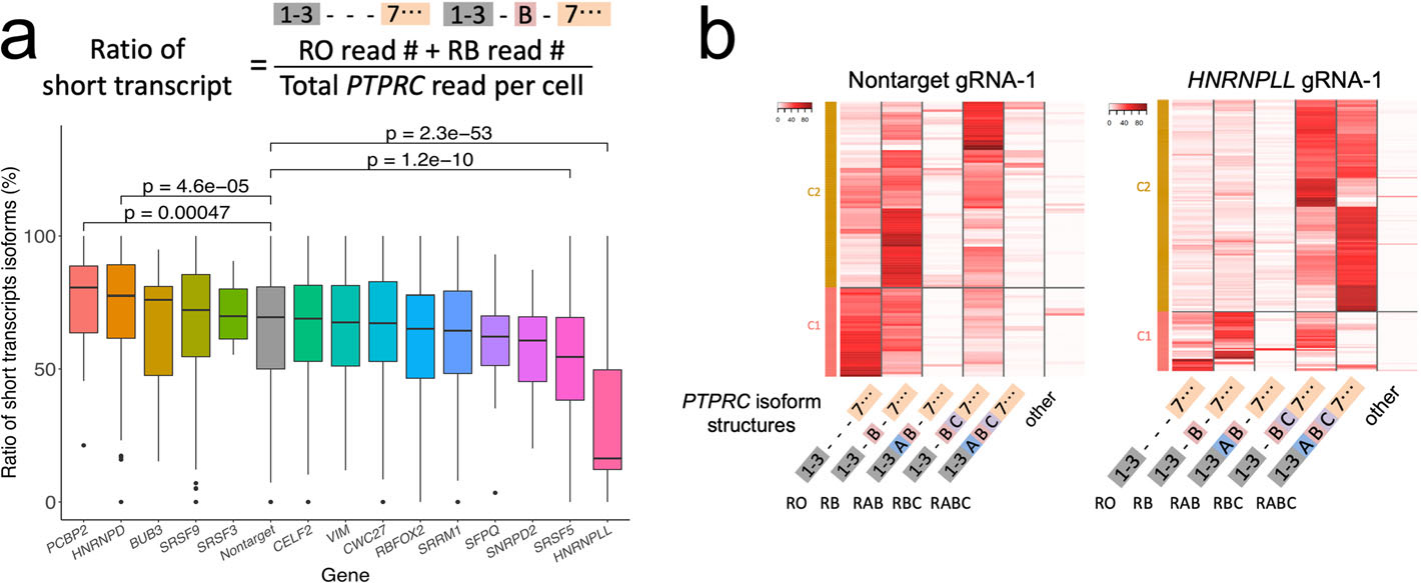
**a** Boxplot showing the ratio of short *PTPRC* transcript isoform (RO and RB) for cells with gRNAs targeting indicated genes. *P* values are calculated in comparison with Nontarget. Genes which have less than three cells with target gRNAs are not shown. **b** Heatmap showing proportion of each transcript isoform (*x*-axis) with each cell (*y*-axis) and clustering based on transcript isoform proportion for cells having indicated gRNA sequence

Next, we conducted long-read sequencing of the *PTPRC* cDNA amplicon from individual cells. To amplify a specific cDNA, we used primer pair of 5′ cDNA adaptor and *PTPRC* exon7 sequences. To filter out PCR artifact, we only used the reads which align to both exon3 and exon7 of *PTPRC*. Overall, 98% of reads covered up to exon1 with the remaining 2% having a truncation as result of early termination of reverse transcription. We clustered individual cells based on the expressed gRNA sequences targeting the different splicing factor genes. We determined the expression and relative ratios of the five most abundant *PTPRC* transcript isoforms for these cells using long-read sequencing. For each long read, the isoform structure was identified by determining aligned bases within each exon. The cell barcode was identified using the sequence matching methodology described earlier. Among the different cells with the CRISPR gene targets versus the negative control, we compared the average ratio of short *PTPRC* isoform category and the fold change differences (Fig. 3A and Additional file 1: Fig S10). The knock-out of *HNRNPLL* and *SRSF5* reduced *PTPRC* exon-skipping events, resulting in lower RO and RB abundance (2.01- and 1.18-fold change respectively). In comparison, the knock-out of *PCBP2* and *HNRNPD* increased exon-skipping events resulting in higher RO and RB abundance (1.15- and 1.16-fold change respectively). Therefore, *HNRNPLL* and *SRSF5* induced exon skipping of *PTPRC*. In contrast, *PCBP2* and *HNRNPD* inhibited exon skipping. Although *HNRNPLL* and *SRSF5* knock-outs inhibited *PTPRC* exon skipping, their isoform expression patterns were significantly different (Additional file 1: Fig S11 and 12). The ratio of RBC was comparable between the two knock-outs but the ratio of RABC was much higher for the *HNRNPLL* knock-out (5.60-fold change, *P* = 5.90e−37). The consequences of *PCBP2* and *HNRNPD* knock-outs were nearly equivalent with respect to the expression of the RB isoform (*P* = 0.1). However, the *PCBP2* knock-out had greater expression of the RO isoform (1.20-fold change, *P* = 0.017) (Additional file 1: Fig S13).

Overall, *HNRNPLL* had the most significant effect on *PTPRC* isoform regulation. This result is concordant with previous reporting that HNRNPLL binds to ESS1 site at *PTPRC* exon4 [18]. We compared the cells expressing the nontarget-1 gRNA versus the cells expressing the *HNRNPLL*-1 gRNA (Fig. 3B). In comparing the cells with nontarget-1 gRNA, the cells expressing the *HNRNPLL*-1 gRNA had a significantly different expression patterns and ratios of *PTPRC* isoforms. These cells had a relatively lower expression of RO and RB isoforms (2.45-fold, *P* = 1.16e−32).

### Confirmation of the result with long-read sequencing by CRISPR RNP experiment

**Fig. 4:**
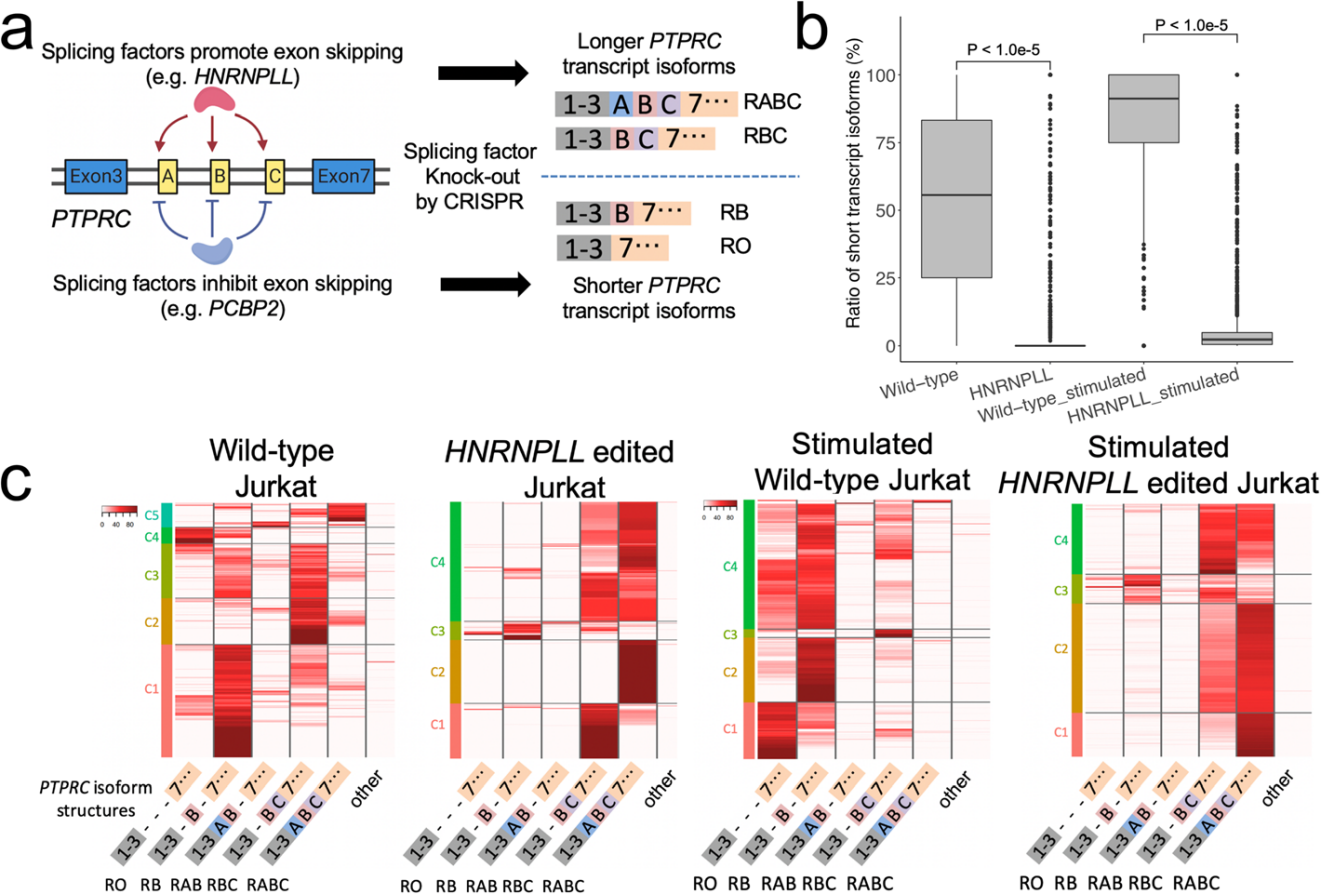
**a** Overview of splicing factors affect alternative splicing. **b** Quantification of short transcript isoform per target gene. For each sample (*x*-axis), the ratio of transcript isoform RO and RB among all *PTPRC* isoforms for cells are shown as box plot. **c** Heatmap showing proportion of each transcript isoform (*x*-axis) with each cell (*y*-axis) and clustering based on transcript isoform proportion for each sample

To verify this result as well as demonstrate that this method translates to another CRISPR delivery method, we selected the *HNRNPLL* gene for an independent knock-out experiment. We used an electroporation approach to introduce the Cas9 ribonucleoprotein (RNP) targeting *HNRNPLL* gene into Jurkat cells (Methods). Six days after electroporation, we prepared single-cell cDNA libraries from the wild-type Jurkat cells and KO pool cells. Subsequently, we performed single-cell short-read sequencing to enumerate RNA expression and long-read sequencing to determine the *PTPRC* isoforms per each cell. Similar to the previous single-cell CRISPR screen result, wild-type cells demonstrated abbreviated transcripts (i.e., RO, RB) resembling the isoform profile of naïve primary T cells, while *HNRNPLL* RNP-treated cells expressed the longer transcript isoforms (i.e., RABC, RBC) (7.35-fold, *P* < 1.0e−5, Fig. 4B, C). When comparing the RNP versus lentivirus-based *HNRNPLL* gRNAs, we observed similar *PTPRC* transcript structures (Figs. 3B and 4C).

This experimental system had an additional functional readout to assess *PTPRC* isoform expression. Exposing Jurkat cells to phorbol 12-myristate 13-acetate (PMA) and ionomycin induces the expression of shorter isoforms of *PTPRC* [19]. Taking advantage of this cellular property, we challenged both CRISPR-treated and wild-type cells with this small molecule combination then subsequently performed single-cell sequencing. Overall, our results showed that PMA and ionomycin stimulation increased the differences in isoform expression between the wild-type cells and *HNRNPLL* RNP-treated cells. Most of the stimulated wild-type cells had RO and RB transcript isoforms. However, the stimulated *HNRNPLL* RNP-treated cells had fewer RO and RB transcript isoforms (10.32-fold, *P* < 1.0e−5, Fig. 4B, C).

### Direct detection of CRISPR-induced *MYL6* transcript isoform perturbation

In addition to *PTPRC*, we analyzed the impact of splicing factors on myosin light chain 6 (*MYL6*) transcript isoforms. Exon6 skipping of *MYL6* is known to be regulated by various splicing factors (Additional file 1: Fig S14A) [1, 20]. We found that *CELF2* targeted cells had a higher percentage of full-length *MYL6* transcript isoforms compared to cells targeted by other splicing factors (1.44-fold, *P* = 1.9e−14, Additional file 1: Fig S14B), indicating that disrupting *CELF2* reduced the occurrence of exon-skipping.

## Discussion

When analyzed with short reads, single-cell CRISPR screens do not verify the edit genotype and miss transcript features due to the limitation on sequence length. Addressing these limitations, our new methodology leverages long-read sequencing technology for single-cell CRISPR analysis. As a result, CRISPR edit genotypes are directly confirmed in the target transcript. Isoform variation and levels of expression become evident at the resolution of individual cells. In addition to the effect of the knock-out of splicing factors on transcript isoform generations, we can assess the transcriptomic result of known exon skipping or intron retention events as shown in Fig. 1. Beyond its application on identifying transcript isoforms, we demonstrated that this approach also enables one to directly assess the presence of CRISPR indels in the transcript. The sequence of expressed guide RNA in each cell, which defines the analysis window for CRISPR indels, enables this analysis. Future work will test if this method can also assess single-nucleotide variants in the transcript.

For this study, we demonstrated that our method can be applied across different CRISPR transduction approaches including plasmid, lentiviral, and RNP delivery. In the case of the RNP method, one can use this method to assess individual or sets of cells directly for the presence of the target alteration. This approach obviates the need for maintaining the expression of a guide RNA within an individual cell for sequencing as is required by other single-cell CRISPR methods.

Our approach can be used with CRISPR base editing methodology. Unlike Cas9 nuclease which induces a complete gene KO, base editors introduce base substitution. However, base editors can induce a range of different base substitutions outside of what was originally planned. This lack of specificity leads to unexpected amino acid substitutions unless there is only one target nucleotide at the site of interest. Combining our long-read methodology with base editors, we can evaluate in a highly scalable and rapid fashion those cells with the intended substitution versus those with an unintentional base change. This feature allows one to determine the phenotypic effects of single base substitution within the target of interest.

## Conclusion

Our approach demonstrates a new methodology for the characterization of CRISPR-induced transcript isoform modifications. We could detect transcript isoform changes induced by disruption of splicing sites or knock-out of splicing factors at the single-cell level. Furthermore, CRISPR-induced mutations could also be detected at the single-cell level. There are many unresolved questions about the biological effect of specific transcript isoform changes or the regulation of various splicing factors. Our method will enable multiplexed analyses of these questions by using multiplexed single-cell CRISPR screens.

## Methods

### Cell culture conditions

HEK293T (ATCC CRL-11268) cells and Cas9-stable HEK293T (T3251; Applied Biological Materials Inc., Richmond, BC, Canada) were maintained in Dulbecco’s modified Eagle’s medium (DMEM) supplemented with 10% fetal bovine serum (FBS). Jurkat (ATCC TIB-152) and Cas9-stable Jurkat (SL555, GeneCopoeia, Inc., Rockville, MD, USA) cells were maintained in Roswell Park Memorial Institute (RPMI) 1640 medium supplemented with 10% FBS. Cells were authenticated based on sequencing analysis. All cells were confirmed by PCR to be free of mycoplasma contamination.

### Transfection and electroporation condition

We used 1.2 × 10^6^ HEK293T cells to transfect the Cas9 expression plasmid (1000 ng) and sgRNA plasmids (1000 ng) using Lipofectamine 2000 (Invitrogen, Carlsbad, CA, USA) according to the manufacturer’s protocol. For electroporation, 2 × 10^5^ Jurkat cells were used. We used 1250 ng of TrueCut™ Cas9 Protein v2 (Invitrogen) and then we used 585 ng of sgRNA which were incubated at room temperature for 10 min before the electroporation to form the RNP complex. RNP was added to Jurkat cells, resuspended in R buffer, and electroporated using Neon electroporation system (1700 V / 20 ms / 1 pulse, Invitrogen). Electroporated cells were transferred to a 24-well plate containing the culture medium. After 6 days of transfection or electroporation, cells were subjected to single-cell library preparation or stimulation.

### Single-cell library preparation

Single-cell cDNA and gene expression libraries are prepared using Chromium Next GEM Single Cell 5' Library & Gel Bead Kit v1.1 or v2 (10X Genomics, Pleasanton, CA, USA) as per the manufacturer’s protocol. The cDNA and gene expression libraries are amplified with 16 and 14 cycles of PCR respectively. The quality of gene expression libraries is confirmed using 2% E-Gel (Thermo Fisher Scientific, Waltham, MA, USA). The libraries are then quantified using Qubit (Invitrogen) and sequenced on Illumina sequencers (Illumina, San Diego, CA, USA).

### Long-read sequencing

From the single-cell full-length cDNA, 25 ng was used to amplify transcripts. Primer sequences: Partial_R1 – CTACACGACGCTCTTCCGATCT, RACK1_ex8 – ACACTCGCACCAGGTTGTCCG, PTPRC_ex7 – CCAGAAGGGCTCAGAGTGGT, MYL6_ex7 - ACACAGGGAAAGGCACGGACTCTGG. KAPA HiFi HotStart ReadyMix (Roche, Basel, Switzerland) was used for amplification. Extension time was 45 s for *PTPRC* and 60 s for other genes. Libraries were prepared with 900 fmol of each amplicon for MinION flow cell FLO-MIN106D (Oxford Nanopore Technologies, Oxford, UK) and Promethion flow cell FLO-PRO002 (Oxford Nanopore Technologies) or 80 fmol for Flongle flow cell FLO-FLG001 (Oxford Nanopore Technologies) using Native Barcoding Expansion and Ligation Sequencing Kit (Oxford Nanopore Technologies) as per the manufacturer’s protocol. Libraries were sequenced on MinION or Promethion over 48 h.

### Lentiviral gRNA library production

The oligonucleotide pool for gRNA library cloning was ordered using IDT oPools Oligo Pools (Coralville, Iowa, USA). Amplified gRNA cassettes were cloned to lentiGuide-Puro (Addgene plasmid #52963) using NEBuilder HiFi DNA Assembly Master Mix (New England Biolabs, Ipswich, MA, USA). Cloned plasmids were purified and electroporated to ElectroMAX Stbl4 competent cells (New England Biolabs).

### Lentivirus production

2.0 × 10^6^ HEK293T cells were plated 24 h prior to transfection. Cells were transfected with lentiviral sgRNA library (2000 ng), psPAX2 (1500 ng, Addgene plasmid #12260), and pMD2.G (500 ng, Addgene plasmid #12259) using Lipofectamine 2000 (Invitrogen) according to the manufacturer’s protocol. The viral supernatant was collected after 48 h of transfection, filtered through a 0.45 μm filter, and used.

### Lentivirus transduction

To 1.0 × 10^5^ Cas9-stable Jurkat, the lentiviral supernatant and 8 μg of polybrene (Sigma-Aldrich, MO, USA) were added and the mixture was centrifuged at 800*g* for 30 min at 32 °Celsius. After that, cell pellets were resuspended to fresh media and plated in a 6-well plate. After 72 h, transduced cells were selected by puromycin (Life technologies, CA, USA).

### Guide RNA sequencing

The sgRNA direct capture was performed using Chromium Next GEM Single Cell 5' Library & Gel Bead Kit v2 (10X Genomics) as previously described [17]. Then, 6 pmol of gRNA scaffold binding primer (oJR160) was added to RT master mix directly before droplet generation. After cDNA amplification, we performed 0.6X left-sided SPRI cleanup reaction for cDNAs and 0.6X–1.8X double-sided SPRI selection for gRNA using SPRIselect (Beckman Coulter Life Sciences, CA, USA). The sgRNA fractions were amplified by primers oJR163 and oJR163 (Additional file 7: Table S6) and sequenced with gene expression library.

### Single-cell transcript isoform analysis

#### Short-read transcripts

Basecalling for 5′ gene expression libraries was performed using cellranger 3.2 (10X Genomics) mkfastq, followed by alignment to reference genome GRCh38, and transcript quantification using cellranger count. In preparation for integrated analysis, the transcript count matrices generated by cellranger were processed by Seurat 3.0.2 [21]. QC filtering removed cells with fewer than 100 or more than 8000 genes, cells with more than 30% mitochondrial genes and cells predicted to be doublets by DoubletFinder [22]. Additionally, any genes present in 3 or fewer cells were removed. Dimension reduction was performed using principal component analysis (PCA) and UMAP with 30 principal components and cluster resolution of 0.8.

#### Long-read transcripts

Basecalling was performed using guppy 4.5.4 [11] and alignment to the reference genome GRCh38 using minimap2 v2.17 [12]. A custom python script utilizing pysam and the exon coordinates for the gene of interest (*RACK1*, *PTPRC*, or *MYL6*) identified reads which span exon1 through exon8 for *RACK1*, exon3 through exon7 for *PTPRC*, or exon5 through exon7 for *MYL6.* For each read, exons which were present in the transcript were identified and recorded: for example 1234-678 indicated a *RACK1* transcript which skips exon 5.

#### Integration of long and short reads

Using knowledge of the adapter/cell barcode/UMI structure in the reads as well as the valid filtered single-cell barcodes from short-read sequencing, the putative long-read barcode is identified. This process involves evaluating the soft-clipped portions of the aligned long reads, which are extracted utilizing the pysam module in a custom python script. A second custom python script used a machine-learning approach to identify the barcode. First, the list of valid short-read barcodes was vectorized using the CountVectorizer function from the Scikit-learn python module, with a kmer length of 8 to create a reference list. For plus strand genes such as PTPRC, the left soft-clipped region of each aligned read was then vectorized in the same way and compared to the created reference using a cosine similarity metric also from Scikit-learn. Similarly, for minus strand genes such as RACK1, the right soft-clipped region of each aligned read was evaluated by matching the reverse-complement of the soft-clipped sequence to the reference list.

For each read, the barcodes with highest cosine similarity to the reference list were evaluated further. Edit distance to each of the top five reference barcodes having a non-zero cosine similarity score was calculated for each 16-bp window across the soft-clipped search sequence. The barcode with lowest edit distance (and in cases of a tie, the highest cosine similarity score) was selected for final filtering. If the sequenced cDNA barcode had an edit distance < 3 from the reference short-read barcode, it was considered a matching barcode; otherwise, the read was not considered a match to any of the short-read barcodes and was excluded from further integrated analysis. Per the 10X single-cell library preparation process, we know that the 10 bp UMI is located directly adjacent to the 16-bp cell barcode. Having established the start position of the best matching barcode as above, the start position of the UMI can be deduced. For reads sharing the same barcode, their 10 bp UMI is evaluated and any exact matches discarded as duplicates. We are unable to use UMI sequences whitelisted from the short reads due to the much sparser gene expression per cell over the entire transcriptome, versus the long reads for which our genes of interest are amplified. Output from the barcode matching script was then summarized using awk and bash commands to provide transcript counts per isoform exon pattern identified from the initial long-read processing for each barcode. A clustered heatmap was generated in R using heatmap.2, cutree, and dendextend, showing the proportion of each isoform per cell barcode. The isoform cluster for each barcode was then added to the Seurat object as a metadata column using AddMetaData, to enable this categorization to be used in subsequent visualization plots.

#### CRISPR-induced mutation analysis

For each gRNA, we set the analysis window as 2 bp around the Cas9 cleavage site. For each aligned long read, we extracted alignment positions and aligned bases within the analysis window. Sequences which perfectly matched the reference sequence were considered wild-type and sequences with insertions, deletions, or base substitutions were assigned as mutations. We performed this analysis for each gRNA for each cell and summarized the mutation rates per gRNA.

### Guide RNA in vitro transcription

sgRNA was in vitro transcribed by T7 RNA polymerase using the MEGAshortscript T7 kit (Invitrogen) according to the manufacturer’s protocol. Templates for sgRNA were generated by extension of two complementary oligo nucleotides. Transcribed RNA was purified by column purification. Purified RNA was quantified by Qubit 4 Fluorometer (Invitrogen).

### Jurkat cell stimulation

2 × 10^6^ Jurkat cells were stimulated with eBioscience™ Cell Stimulation Cocktail (Invitrogen) using half of the recommended concentration in a 6-well plate for 24 h. Stimulated cells were subjected to single-cell library preparation.

## Supplementary Information

**Additional file1: Fig S1.** Guide-RNA designs targeting splicing acceptor of RACK1. **Fig S2.** Distribution of edit distance of cell barcode from each long-read sequence compared to matching cell barcode from whitelist. **Fig S3.** UMI distribution per cells in wild-type HEK293T cells. **Fig S4.** RACK1 transcript isoform composition of wild-type HEK293T cells. **Fig S5.** Quantification of RACK1 expression level for each cluster. **Fig S6.** Volcano plot differentially expressed gene between cells with full-length or exon5 skipped RACK1 transcript. **Fig S7.** UMAP plots colored by transcript isoform based cell clusters. **Fig S8.** Structure of major transcript isoforms of PTPRC. **Fig S9.** Validation of gRNA identified by short-read sequencing and its consequential CRISPR editing by long-read sequencing per individual cell. **Fig S10.** Average short isoform proportion per targeted genes. **Fig S11.** Transcript isoform proportion for cells having nontarget gRNA sequence. **Fig S12.** Transcript isoform proportion for cells having HNRNPLL and SRSF5 targeting gRNA sequence. **Fig S13.** Transcript isoform proportion for cells having HNRNPD and PCBP2 gRNA sequence. **Fig S14.** Effect of various splicing factor KO on MYL6 alternative splicing.

**Additional file 2: Table S1.** Sequencing metrics.

**Additional file 3: Table S2.** Single-cell sequencing quality metrics

**Additional file 4: Table S3.** List of gRNAs.

**Additional file 5: Table S4.** List of differentially expressed gene between cells with full-length or exon5 skipped *RACK1* transcript.

**Additional file 6: Table S5.** gRNAs for single-cell CRISPR screen.

**Additional file 7: Table S6.** List of oligo nucleotides for gRNA capture.

**Additional file 8: Table S7.** Mutation rate detected from long-read sequencing for each gRNA target.

**Additional file 9.** Review history

## Data Availability

The sequencing data have been deposited in the NCBI Sequence Read Archive database under the accession number PRJNA708300 [23]. Scripts for analysis are publicly available on github (https://github.com/sgtc-stanford/scCRISPR) [24] and zenodo (https://zenodo.org/badge/latestdoi/365008149) [25] under MIT license.
