# Supplementary material for: Single-cell characterization of CRISPR-modified transcript isoforms with nanopore sequencing
Source: Genome Biol. 2021 Dec 6;22:331. doi: 10.1186/s13059-021-02554-1 (PMC8647366; doi:10.1186/s13059-021-02554-1)

SUPPLEMENTARY MATERIALS

Single cell characterization of CRISPR-modified transcript isoforms with nanopore sequencing

Authors:

Heon Seok Kim[1], Susan M. Grimes[1], Anna C. Hooker[1], Billy T. Lau[1], Hanlee P. Ji[1]

Institutions:

[1] Division of Oncology, Department of Medicine, Stanford University School of Medicine, Stanford, CA, United States

Corresponding author:

Hanlee P. Ji

Email: genomics_ji@stanford.edu

CCSR 1115, 269 Campus Drive, Stanford, CA-94305, USA

Fig S1. Guide-RNA designs targeting splicing acceptor of *RACK1*. Red and blue bases indicate protospacer adjacent motif (PAM) and splicing acceptor sites respectively.

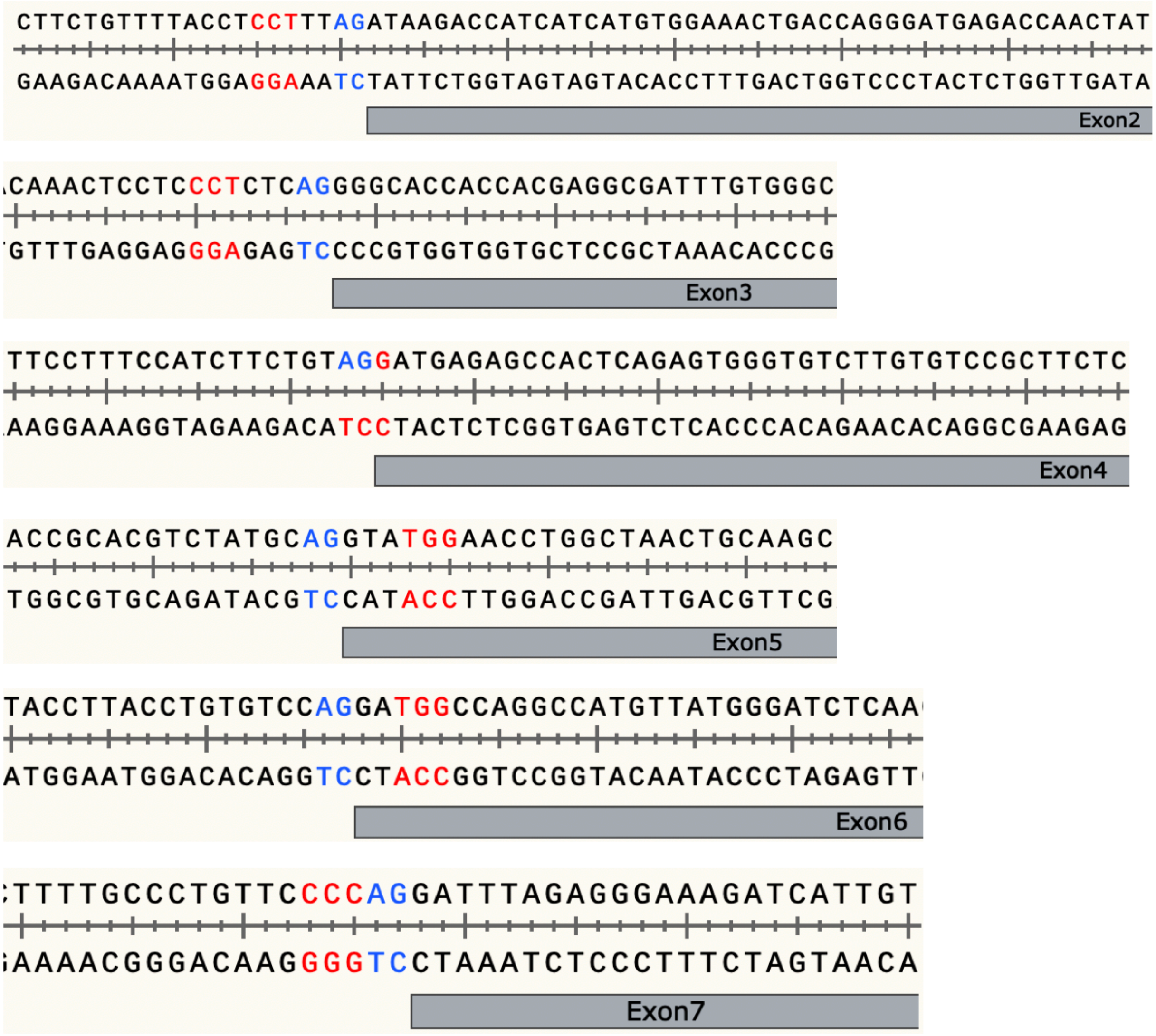

Fig S2. Distribution of edit distance between the cell barcode from each long-read sequence and the matched barcode from the cellranger whitelist. Reads with edit distance >3 (red line) are excluded from the analysis.

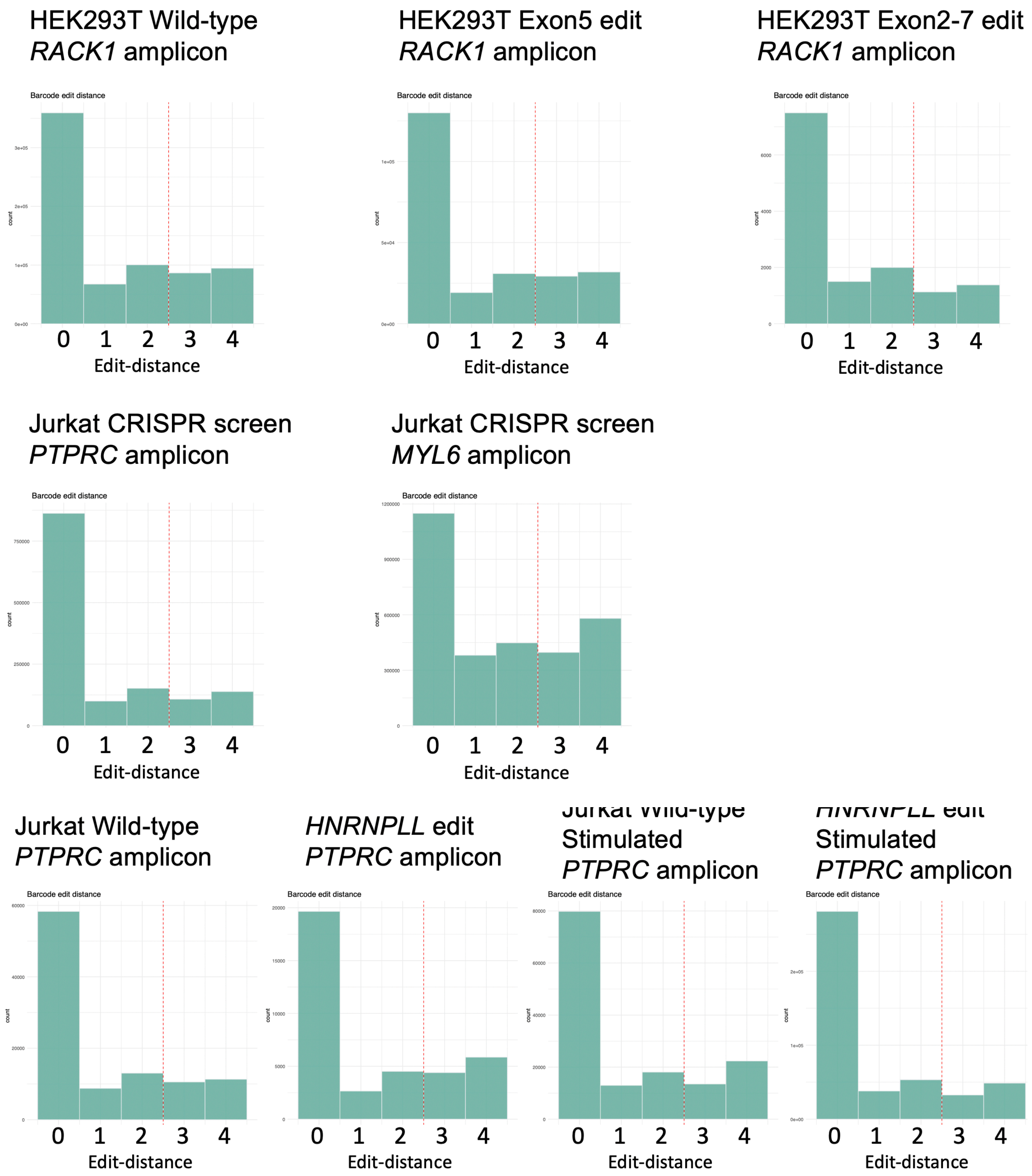

Fig S3. UMI edit distance distribution for a representative sample of cells in the wild-type HEK293T sample. Edit distance between all UMIs in each cell are shown. Cell barcode and read counts per cell are noted.

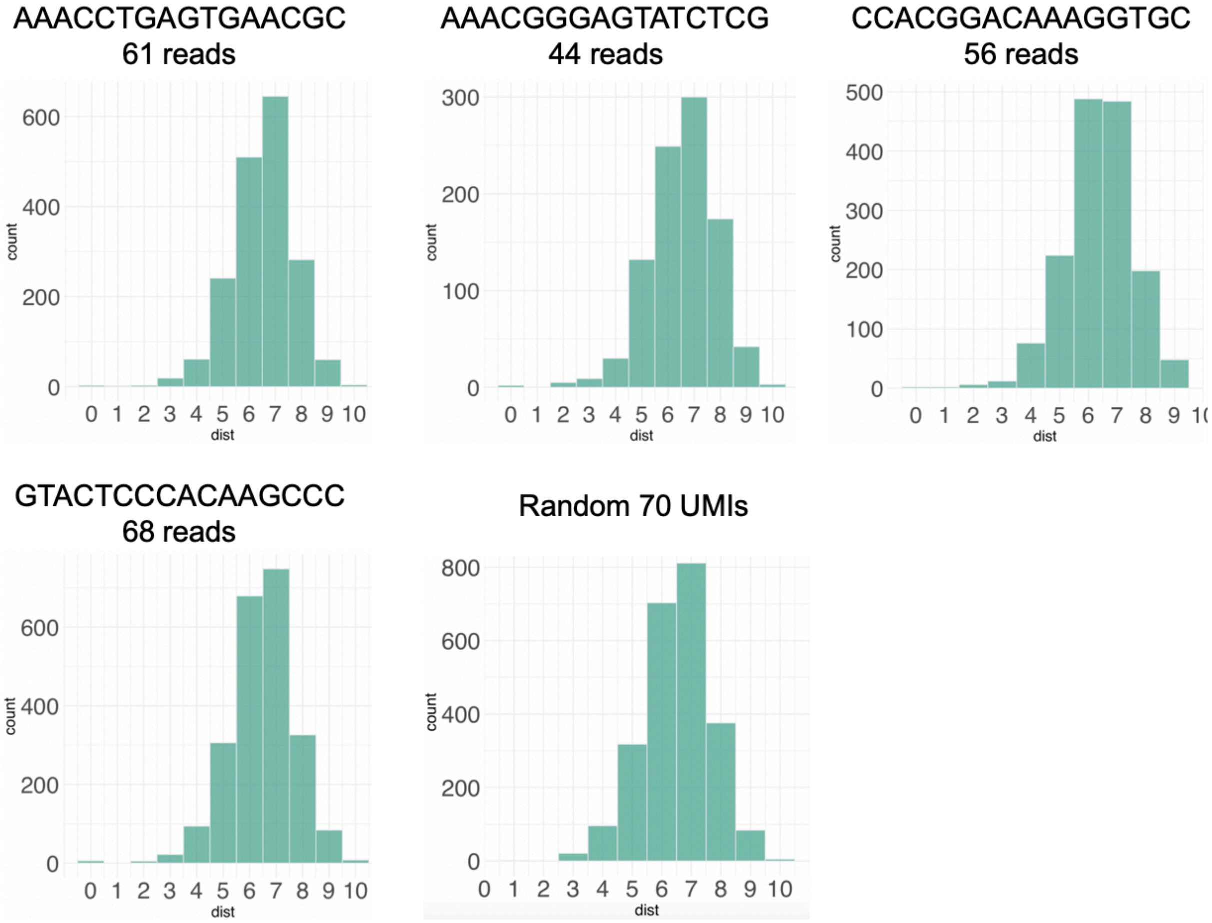

Fig S4. *RACK1* transcript isoform composition of wild-type HEK293T cells. Heatmap showing proportion of each transcript isoform (x-axis) with each cell (y-axis) and clustering based on a given mRNA transcript isoform proportion.

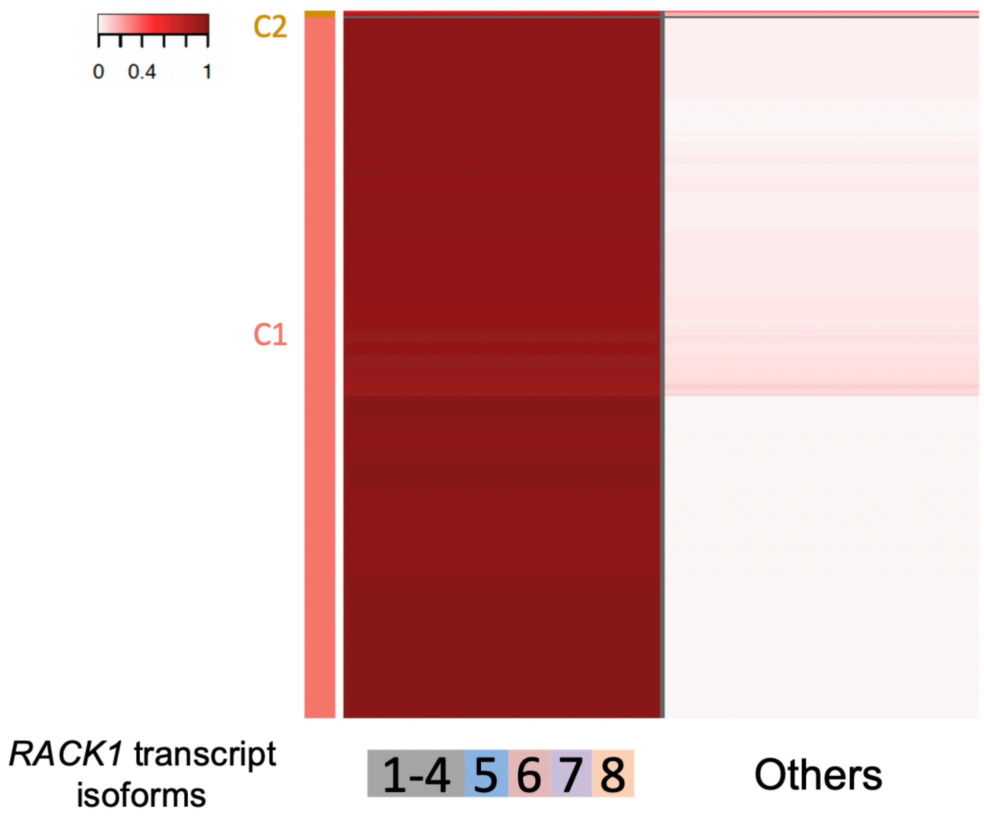

Fig S5. Quantification of *RACK1* expression level for each cluster. Violin plot showing the expression level of *RACK1* in each cluster. P values are calculated in comparison with cluster2. (two-sided t-test)

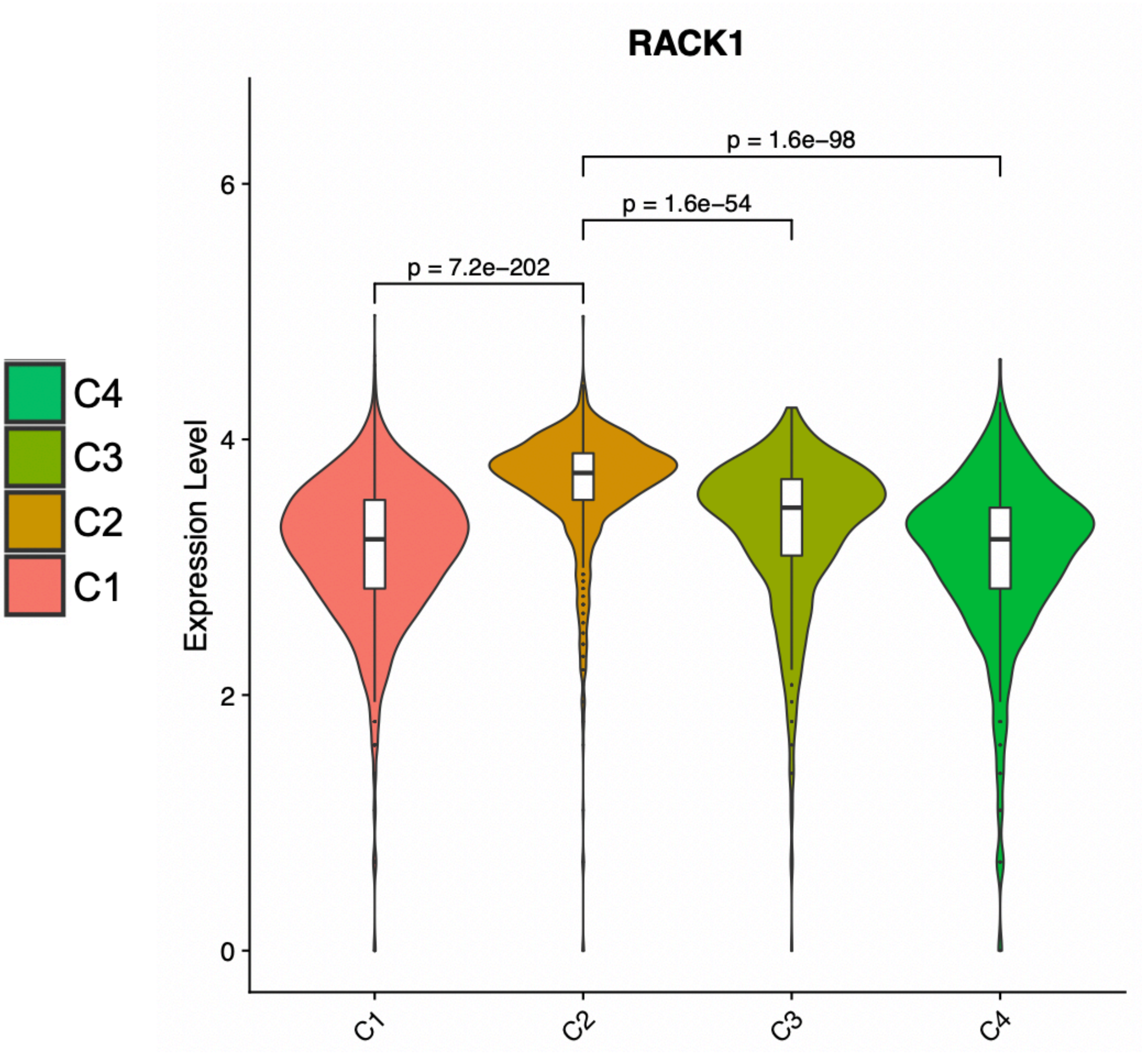

Fig S6. Volcano plot differentially expressed gene between cells with full-length or exon5 skipped RACK1 transcript.

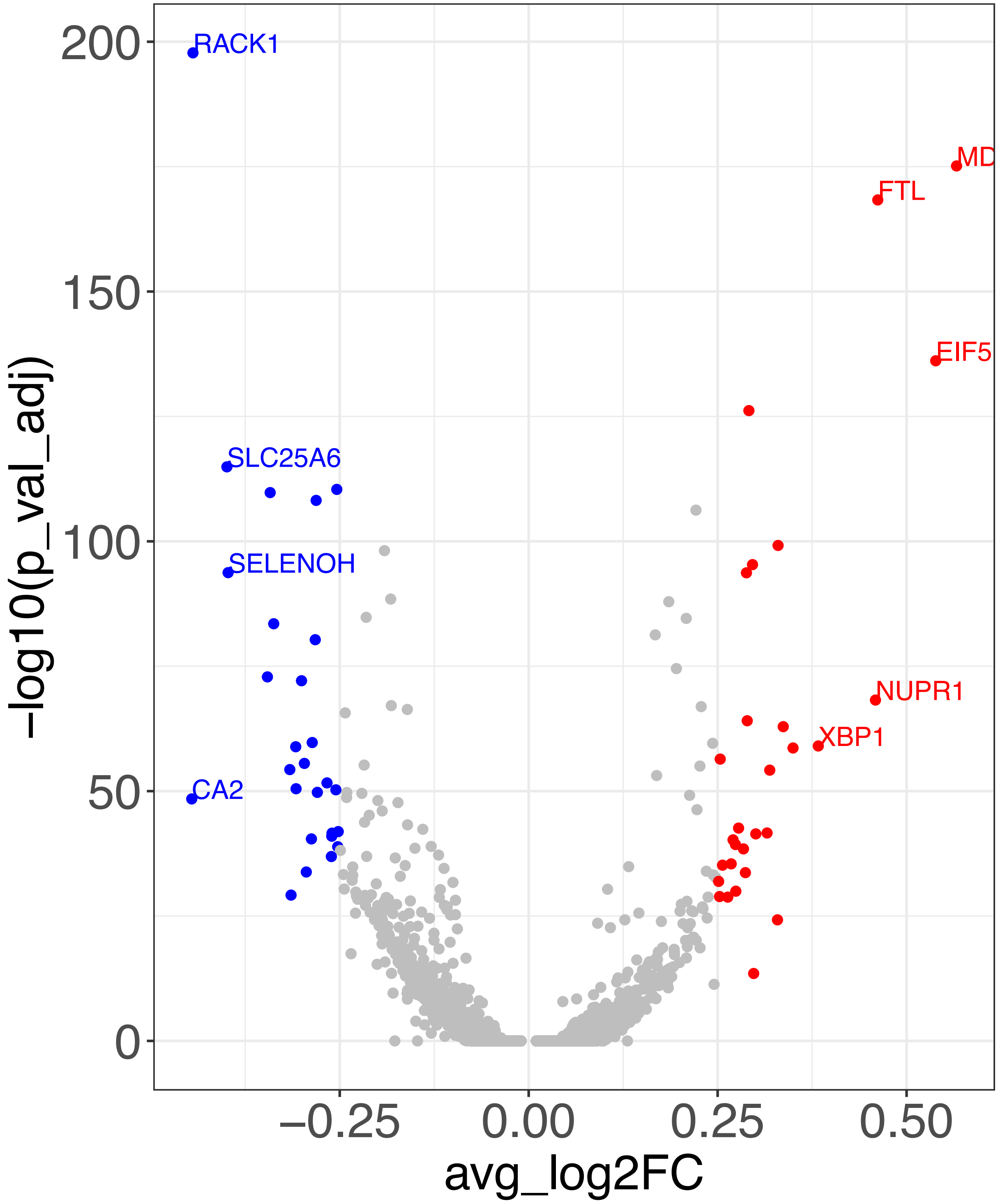

Fig S7. UMAP plots colored by transcript isoform based cell clusters.

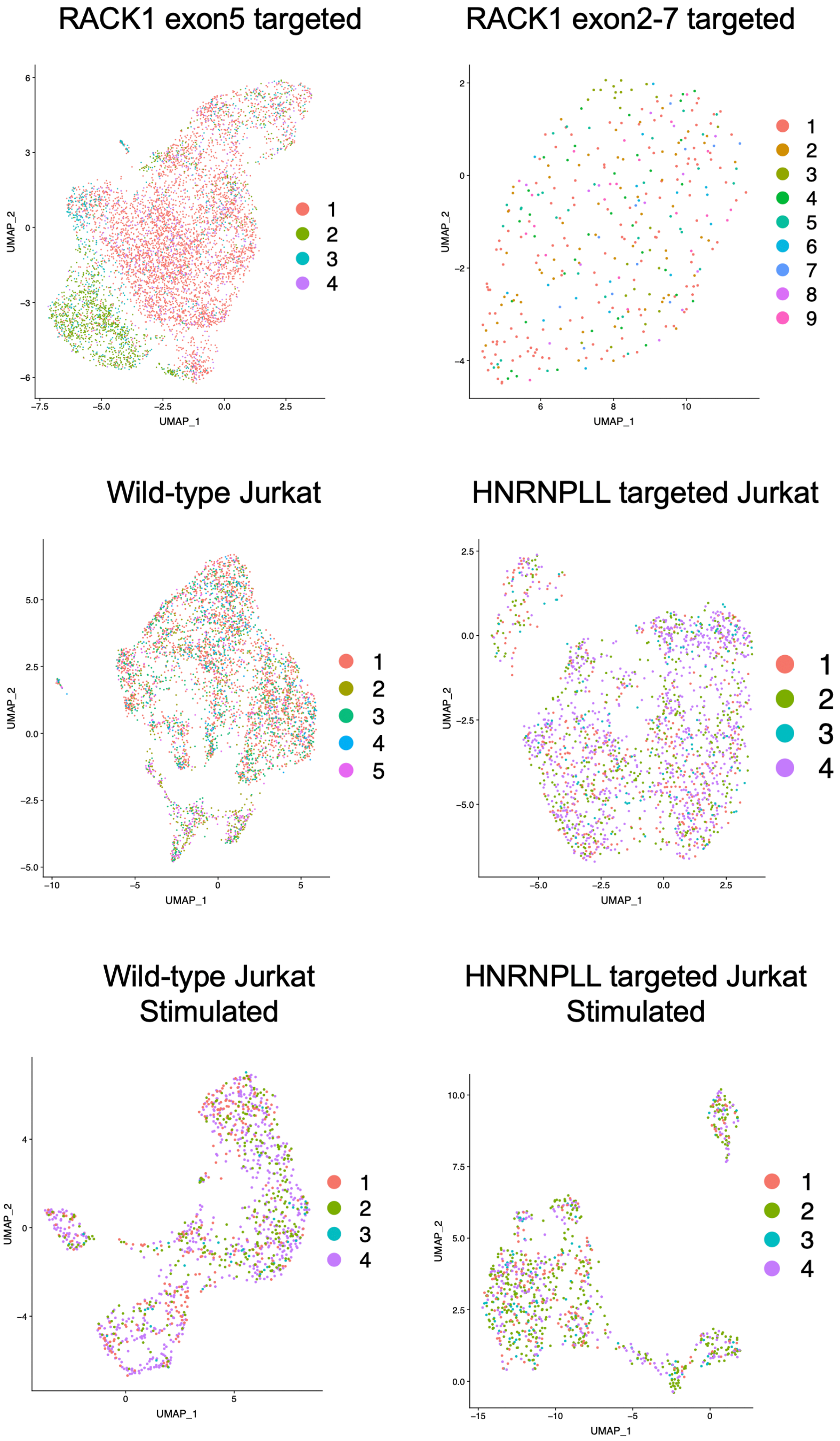

Fig S8. Structure of major transcript isoforms of *PTPRC*. Structures of major 5 transcripts isoform of *PTPRC.* Exon3 to 7 are highlighted.

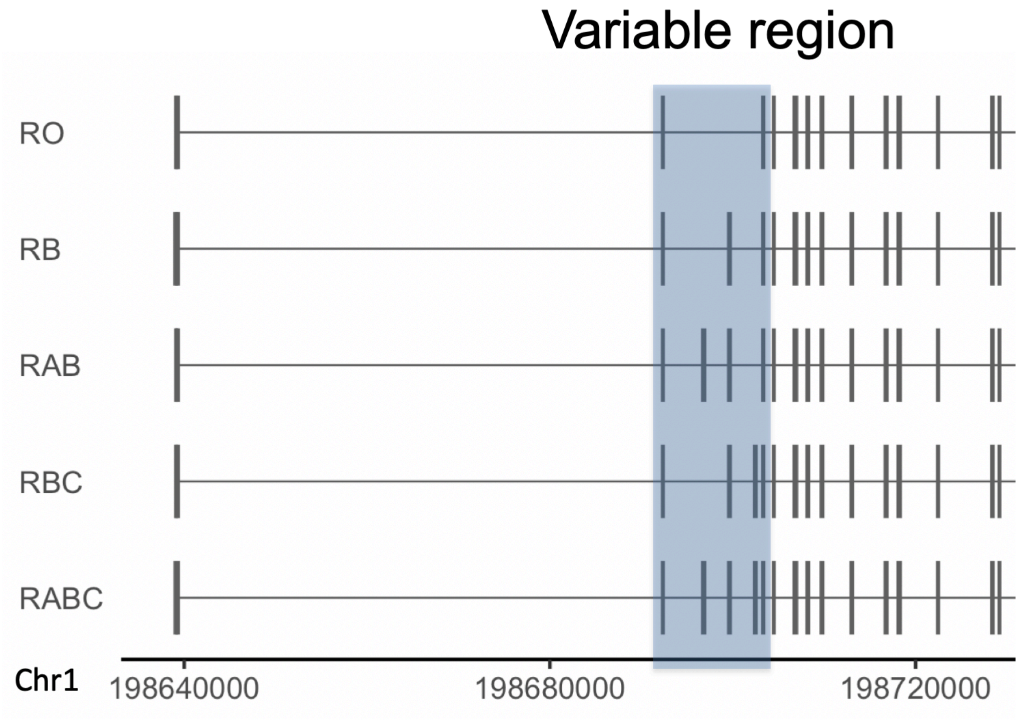

Fig S9. Validation of gRNA identified by short-read sequencing and its consequential CRISPR editing by long-read sequencing per individual cell. (a) Scheme of the experiment. Ad/CB: Sequencing/10x adaptors and cell barcode sequences.

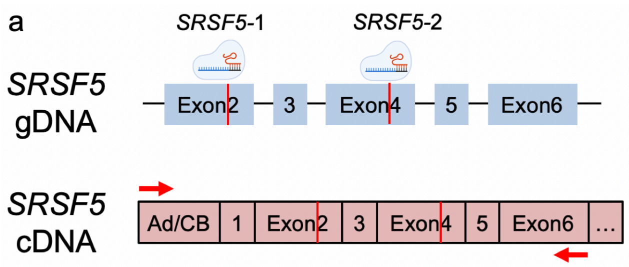

Fig S10. Average short isoform proportion per targeted genes. Heatmap showing the fold change of short *PTPRC* transcripts (RO and RB) proportion per cells having indicated gene targeting gRNA compared to nontarget cells.

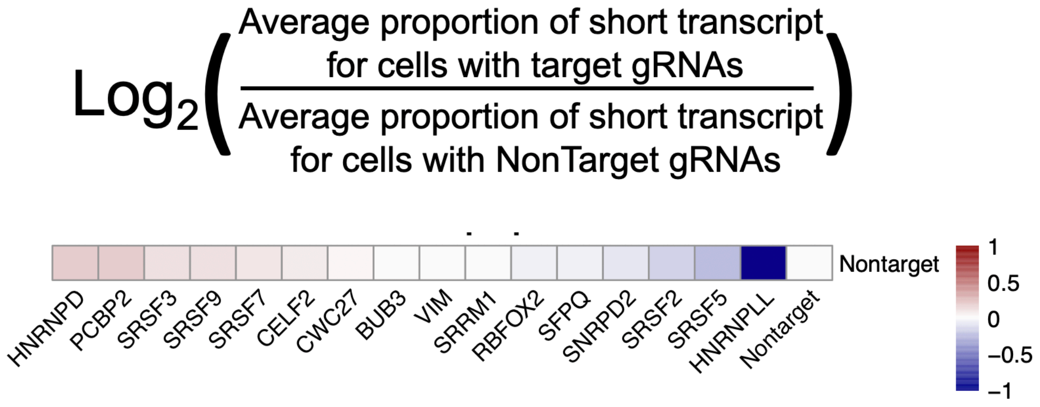

Fig S11. Transcript isoform proportion for cells having nontarget gRNA sequence.

Heatmap showing proportion of each transcript isoform (x-axis) with each cell (y-axis).

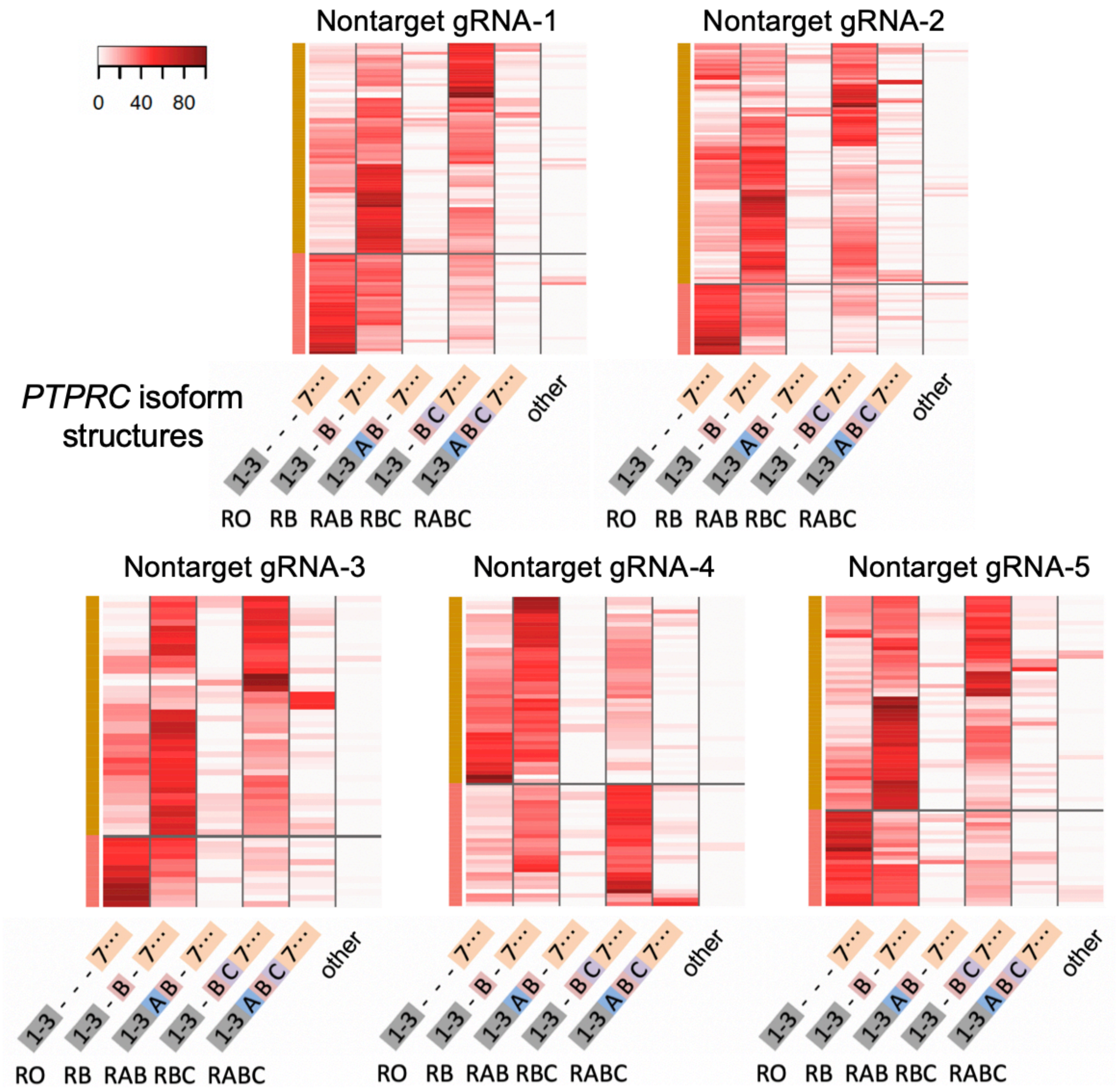

Fig S12. Transcript isoform proportion for cells having *HNRNPLL* and *SRSF5* targeting gRNA sequence. (a) Heatmap showing proportion of each transcript isoform (x-axis) with each cell (y-axis). (b, c) Boxplot showing the ratio of each of long *PTPRC* transcript isoform (RBC and RABC) for cells with the CRISPR target genes.

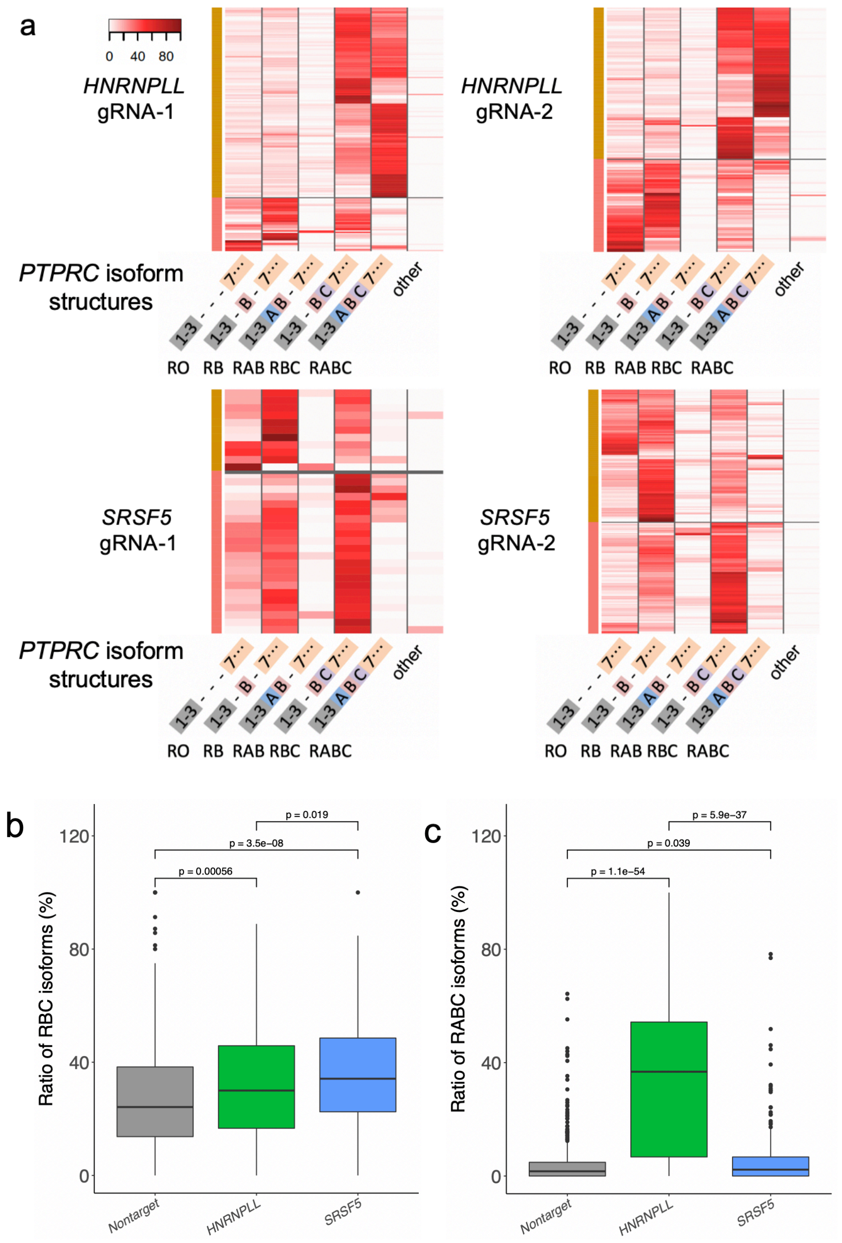

Fig S13. Transcript isoform proportion for cells having *HNRNPD* and *PCBP2* gRNA sequence. (a) Heatmap showing proportion of each transcript isoform (x-axis) with each cell (y-axis). (b, c) Boxplot showing the ratio of each of short *PTPRC* transcript isoform (RO and RB) for cells with the CRISPR targeted genes.

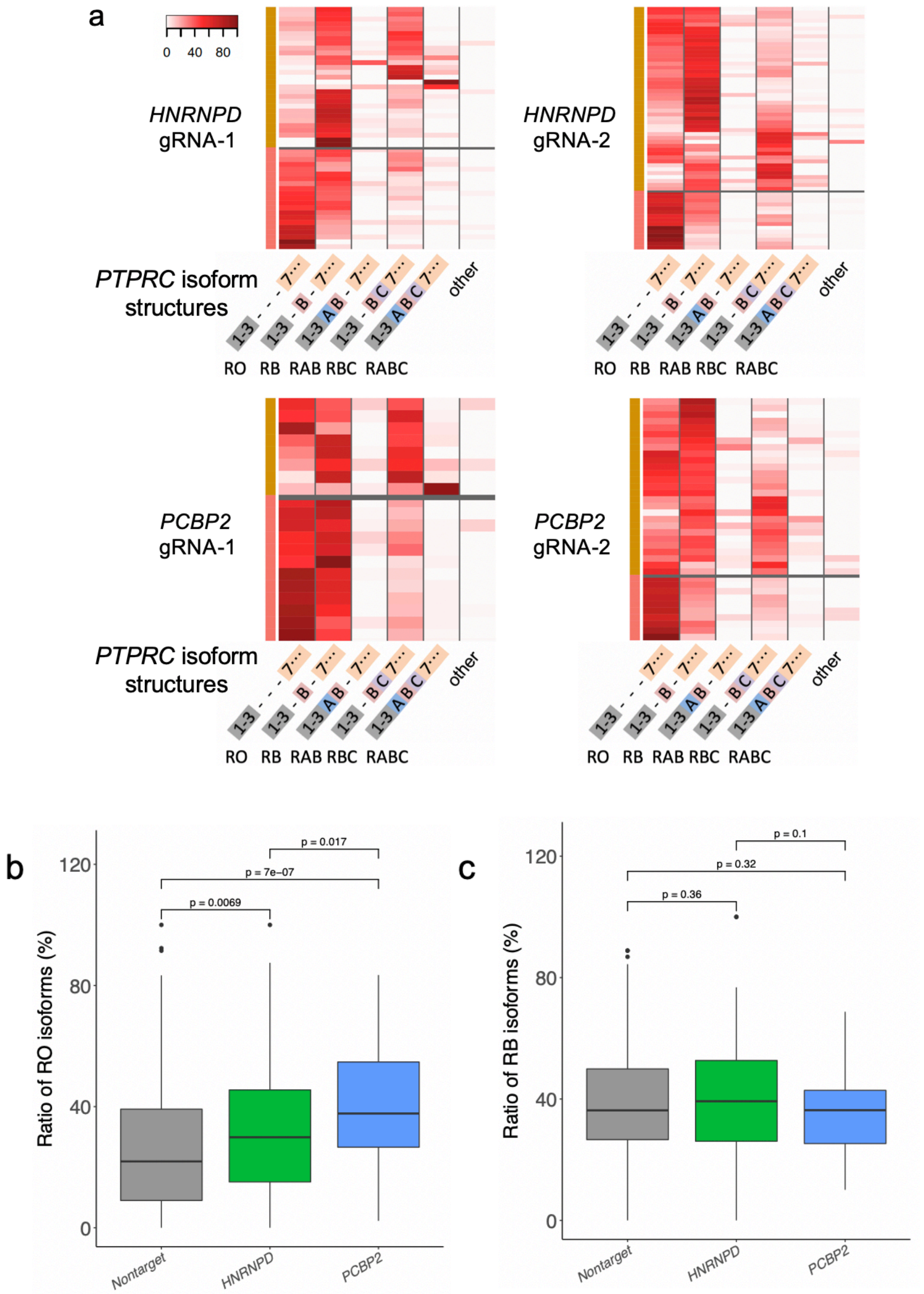

Fig S14. Effect of various splicing factor KO on *MYL6* alternative splicing. (a) Structure of major transcript isoforms of *MYL6.* (b) Boxplot showing the ratio of *MYL6* transcripts with exon6 skipping for cells with gRNAs targeting indicated genes.

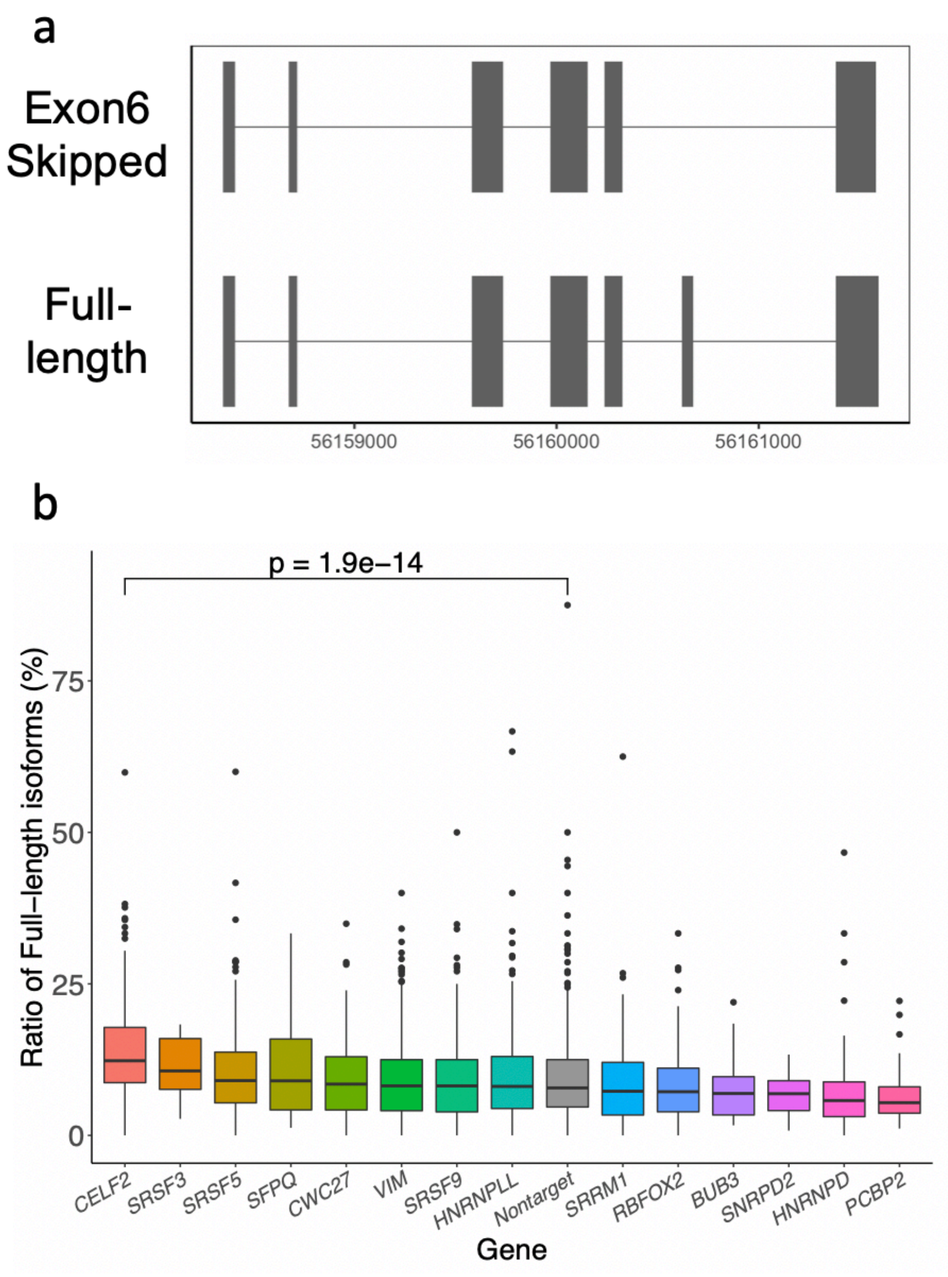

Supplement: Supplementary file 1 — Additional file1: Fig S1. Guide-RNA designs targeting splicing acceptor of RACK1. Fig S2. Distribution of edit distance of cell barcode from each long-read sequence compared to matching cell barcode from whitelist. Fig S3. UMI distribution per cells in wild-type HEK293T cells. Fig S4. RACK1 transcript isoform composition of wild-type HEK293T cells. Fig S5. Quantification of RACK1 expression level for each cluster. Fig S6. Volcano plot differentially expressed gene between cells with full-length or exon5 skipped RACK1 transcript. Fig S7. UMAP plots colored by transcript isoform based cell clusters. Fig S8. Structure of major transcript isoforms of PTPRC. Fig S9. Validation of gRNA identified by short-read sequencing and its consequential CRISPR editing by long-read sequencing per individual cell. Fig S10. Average short isoform proportion per targeted genes. Fig S11. Transcript isoform proportion for cells having nontarget gRNA sequence. Fig S12. Transcript isoform proportion for cells having HNRNPLL and SRSF5 targeting gRNA sequence. Fig S13. Transcript isoform proportion for cells having HNRNPD and PCBP2 gRNA sequence. Fig S14. Effect of various splicing factor KO on MYL6 alternative splicing. [file 13059_2021_2554_MOESM1_ESM.pdf]
