# Supplementary material for: Single-cell characterization of CRISPR-modified transcript isoforms with nanopore sequencing
Source: Genome Biol. 2021 Dec 6;22:331. doi: 10.1186/s13059-021-02554-1 (PMC8647366; doi:10.1186/s13059-021-02554-1)
Supplement: Supplementary file 9 — Additional file 9. Review history [file 13059_2021_2554_MOESM9_ESM.docx]

Review History

**First round of review**

**Reviewer 1**

**Were you able to assess all statistics in the manuscript, including the appropriateness of statistical tests used?**

**No**

**Were you able to directly test the methods?**

No

**Comments to author:**

Overall View:

This study is novel in its combination of single-cell CRISPR with long-read sequencing, an exciting step towards establishing a methodology for high-throughput study of isoform splicing at a cellular level. I believe this work to be suited to the high-quality standard by which is expected of Genome biology. However, some edits towards improving the manuscript for publication is still required. Here are some of my suggestions to the authors:

Major comments.

Comments with regards to Nanopore-sequencing of full-length cDNA library and demultiplexing the resulting data:

During RT and subsequent steps in the generation of full-length libraries, you end up with a lot of (in a lack of a better word) "junk" such as degraded ambient mRNA transcript and many different forms of RT/PCR artefacts. This, in addition to the low quality reads you get from Nanopore-sequencing and its low-throughput capabilities compared to short-read sequencers, makes the profiling of full-length transcripts challenging. Authors overcome these issues by targeted amplification of genes/exons of interest for long-read sequencing. The results look great, but I would like the authors to clarify the following:

* PCR settings used for enrichment of RACK1 and PTPRC, were the same settings used? Or slightly modified to adjust for gene length?

* Clarification on the Nanopore sequencing metrics provided in Supplementary Table 1.

o For aligned reads with RACK1 exon 1&8, and PTPRC exon 3&7, are these values derived as a percentage of all reads, or only from reads that aligned to RACK1 and PTRPC respectively?

o For PTPRC, how often did the authors find reads aligned to more than just exons 3-7?

To first filter for transcripts that align to the outer exon boundaries of interest, prior to demultiplexing, is an excellent strategy for filtering out junk transcripts and improving computational efficiency of cell demultiplexing. The use of "soft-clipped region of the aligned read" from minimap2 to extract Cell barcode/UMI data for demultiplexing is interesting. I would like the authors to clarify further the following:

* Is the barcode whitelist used for cell demultiplexing the entire ~650,000 10X 5' chromium cell barcodes, or only the CellRanger / EmptyDrop filtered barcodes?

* Was there any other filtering done? Did the authors confirm the length of the filtered transcripts are roughly as expected of assigned exon alignments? I expect there still to be significant number of PCR artefacts that might reduce the accuracy of the counts generated.

Can the following text on page 4 please be further clarified?

"Using long read sequencing of target cDNAs, we characterized the transcript isoform diversity in each individual cell. Prior to this quantification step, the 10bp UMI sequence immediately following each cell barcode was reviewed. For any given set of reads that had the same barcode and UMI, these duplicate sequences were discarded."

This is not a trivial task and no mention of UMI count analysis is given in the methods section.

With regards to direct detection of CRISPR induced indel mutations using single cell long read sequencing.

* I think the authors should consider slightly altering the language for this section. Being able to accurately identify SNV from single-cell datasets using long-read sequencer is not trivial, especially without the implementation of a Cellbarcode/UMI consensus forming pipeline. Figure 2b illustrates this point, significant frameshifts are required to align transcripts towards the points of interest. If authors want to insist on this claim, further analysis might be required, and perhaps orthogonal experiments to really look into the accuracy of calling SNVs.

* Authors should add in methods section on how exactly the mutation rates from long-read sequencing data for each gRNA target was calculated (Explain more on the method used to generate supplementary Table 6).

Minor comments:

The PTPRC biology text needs to be refined, how and when T-cells and NK cells express CD45 isoforms is a bit complicated. Authors should perhaps use naïve vs activated/memory T-cells CD45 isoform status as an example for the important biological implications of having the means of studying PTPRC isoforms.

With regards to the comparison of transcriptome between RACK1 CRISPR edited clusters (Fig1c-d): Perhaps the authors should provide a UMAP (generated by short-read data) colour/labelled by isoform profiles C1-C9, so the viewers can visualise the global transcriptional changes are.

Authors might want to give 1 sentence background on the PTPRC isoform profile of Jurkat cells.

For the comparison of gRNA HNRNPLL vs standard Jurkat (Figures 4 & 5), I would have liked to see the scRNA-seq data (UMAPs) but labelled by isoform splice profile. Or perhaps there is no global transcriptional changes? Also, for Figure 4c, it's hard to directly compare the 4 Jurkat profiles by comparing 4 different heatmap figure, though I understand this might be given the nature of the data.

With regards to Jurkat PTPRC isoform splicing experiments.

Authors demonstrate the utility of their method by showing how the isoform splicing profile of PTPRC can be studied following genetic perturbations of splice factors. The splicing of CD45 is a surrogate of many key functional effector properties of T-cells, and so this application would attract the interest of the immunology community.

I believe a killer experiment would be to FACS sort PBMC T-cells into fractions of CD45RA+CDRO- , CD45RA-CD45RO+, and naïve B-cells (CD45ABC), to then be processed using authors methodology. This would act as great orthogonal validation of their isoform results (protein matching PTPRC gene profile), a result that an immunologist with limited technical expertise of single-cell methodologies will get excited about. Though I understand this would lack the CRISPR component thus acts more towards evalutating the robustness of the isoform splicing analysis pipeline.

**Reviewer 2**

**Were you able to assess all statistics in the manuscript, including the appropriateness of statistical tests used?**

**No**

**Were you able to directly test the methods?**

No

**Comments to author:**

In this study, Kim et al assess how gene edits at splicing sites and gene knockouts of splicing factors contribute to the alternative splicing of several genes. Their experiments combine single cell library preparation with long-read (nanopore) sequencing to quantify CRISPR-modified transcript structure. The authors first present how gene edits near the splice junctions of RACK1 exons affect transcript structure, quantified via targeted nanopore cDNA sequencing. Having demonstrated initial feasibility, the authors then target splicing factors and evaluated their impact of the isoform diversity generated by PTPRC transcription.

Although their approach interesting, my enthusiasm for the work is limited due to concerns regarding the experimental quality and presentation of their work. In its current form, the data is not adequately described to enable interpretation of the experiments. In addition, several of the gene editing experiments rely on the activity of single CRISPR guide RNAs at a target site which is inadequate to make definitive comparisons.

Major concerns:

1. Several recent studies have described issues regarding errors in single-cell barcode and UMI sequencing with nanopore (e.g. scCOLOR-seq, Philpott et al., Nature Biotechnology, 2021). The authors should use their short-read sequencing to quantify the long-read error rates and provide convincing analysis that their cosine similarity method is accurately able to call barcodes and UMIs. Some parts of the manuscript (e.g. p. 4, lines 21 - 25) hint at this type of analysis but no clear metrics are provided.

2. The authors emphasize that their method is able to capture the CRISPR-induced mutations at splice junctions but do not show indel data. This is puzzling. To me, the obvious analysis is to correlate the indels with the changes in splicing. Was this done? In particular, did some indels affect splicing more than others? Some evidence is shown in Figure 2B, but given its centrality to the scientific questions being asked, this should be done in a systematic and quantitative fashion.

3. (Figure 1B) The authors do not make it clear on the specific location of guides targeting splice acceptor sites relative to the proximal splice junction. It would be helpful to know if indels were more often located within the introns or exons. Also, does the label Exon1 indicate the entire boxed region? This figure is confusing and needs improvement for clarity.

4. (Figure 1C) It remains unclear why there are two different clusters in Figure 1C that shows a high preference for full length RACK1 transcripts, but only a single cluster in Figure 1D. The heterogeneity of transcripts that express full length RACK1 and transcripts that skip exon 5, seems to be lost. Can the authors elaborate on why this may be?

5. (Figure 1) - The authors do not describe the overall quality of the single cell assays. Line 15 on page 15 does mention that 'any genes present in 3 or fewer cells were removed' which gives an idea of the lower limit of the filtering performed, however it remains unclear as to what portion of single cells met this criterion. Furthermore, for each assay, it would be helpful to know how many unique reads (as a minimum) were acquired from a single cell to support each isoform.

6. (Figure 2C) - It is difficult to interpret why a single guide for each targeted exon outperform the other two guides in terms of efficiency. It would be helpful to know if the affect can be reproduced by two other guides with similar efficiency or have an explanation as to why the experiment would be limited single targeting guide.

7. (Figure 2C) - Would it be possible to repeat this experiment with 3 different gRNAs that successfully generate indels? It might be misleading to only interpret data generated from a single cutting guide. It might also be useful for the authors to comment on any observable trends in successful guide location, relative to the splice junction.

8. (Figure 2) - For the single cell RBP-knockout experiment with nanopore sequencing - it remains unclear what the criteria were for successful UMI matching. For example, what were the mean number of mismatches per UMI to reference. What were the maximum number of mismatches tolerated?

9. (Figure 3A) - It would be useful to better understand why SRSF5 and HNRNPLL knockdowns induced significant shifts in PTPRC isoform generation. Is it possible to assess the putative binding of targeted splicing factors to PTPRC transcripts using motif prediction? Can the authors elaborate on why these genes were chosen for isoform quantification?

10. (Figure 3A) - If eCLIP (or similar data) is available, it would be very useful to know if there is experimental evidence for the direct binding of the targeted RBP splicing factors to PTPRC transcripts. This information would be particularly useful in distinguishing direct interaction of splicing factors to transcripts versus indirect interactions through other RBP intermediates.

11. (Figure 3) - Why were these 14 splicing factors were chosen to be targeted? Given the large number of splicing factors, this choice should be justified.

Minor concerns:

1. (Figure 1B) - The gene model of RACK1 is presented in 3'-5' orientation and is confusing. This can be switched.

2. (Figure 1B) - The red arrows, we interpret as to be pointing to the splice acceptor sites, should be labeled clearly.

3. (Figure 2B) - We observed a comparative lack of positive-stranded reads with SRSF-2. It is unclear if this is due to sampling error and should be clarified.

4. (Supplementary Table 3) - It would be useful to plot these data as a volcano plot, so that the reader can quickly access the number of differentially expressed genes per experiment.

5. (Figure 4) - Can the authors please elaborate as to why electroporation with Cas9 RNPs were used in this experiment and not guide transduction into stably expressed Jurkat cells?

**Authors Response**

**Point-by-point responses to the reviewers’ comments:**

Reviewer #1

Overall View: This study is novel in its combination of single-cell CRISPR with long-read sequencing, an exciting step towards establishing a methodology for high-throughput study of isoform splicing at a cellular level. I believe this work to be suited to the high-quality standard by which is expected of Genome biology. However, some edits towards improving the manuscript for publication is still required. Here are some of my suggestions to the authors:

*We thank the reviewer for the positive feedback. We have addressed all questions and points.*

Major comments.

*Comments with regards to Nanopore-sequencing of full-length cDNA library and demultiplexing the resulting data:*

*During RT and subsequent steps in the generation of full-length libraries, you end up with a lot of (in a lack of a better word) "junk" such as degraded ambient mRNA transcript and many different forms of RT/PCR artefacts. This, in addition to the low quality reads you get from Nanopore-sequencing and its low-throughput capabilities compared to short-read sequencers, makes the profiling of full-length transcripts challenging. Authors overcome these issues by targeted amplification of genes/exons of interest for long-read sequencing. The results look great, but I would like the authors to clarify the following:*

• PCR settings used for enrichment of RACK1 and PTPRC, were the same settings used? Or slightly modified to adjust for gene length?

*The PCR settings were generally the same between the two amplicons. The only difference was the extension time. Extension time for RACK1 was 60s and PTPRC was 45s. We have added these details to the Methods section on Page 14.*

*“Extension time of was 45s for PTPRC and 60s for other genes.”*

• Clarification on the Nanopore sequencing metrics provided in Supplementary Table 1. For aligned reads with RACK1 exon 1&8, and PTPRC exon 3&7, are these values derived as a percentage of all reads, or only from reads that aligned to RACK1 and PTRPC respectively?

*We used a calculation based on the percentage of all reads. We revised the table to provide this information with the following note.*

*“**** Percentages are calculated based on #Reads.” (Supplementary Table 1)*

• For PTPRC, how often did the authors find reads aligned to more than just exons 3-7?

*From the sequencing of the PCR amplification products, we used a partial R1 primer which is located at the 5’ end of the cDNA and PTPRC exon7 binding primer. To address reviewer’s comment, we counted the number of transcripts which align to PTPRC exon1 and 2. Exon1 and exon2 were included for 97.9% and 99.9% of transcripts respectively. We detected an early termination of reverse transcription for some transcripts (~2%) which result in loss of exon1. We have provided this result on Page 9.*

*“Next, we conducted long read sequencing of the PTPRC cDNA amplicon from individual cells. To amplify a specific cDNA, we used primer pair of 5’ cDNA adaptor and PTPRC exon7 sequences. To filter out PCR artifact, we only used the reads which align to both exon3 and exon7 of PTPRC. Overall, 98% of reads covered up to exon1 with the remaining 2% having a truncation as result of early termination of reverse transcription.”*

*To first filter for transcripts that align to the outer exon boundaries of interest, prior to demultiplexing, is an excellent strategy for filtering out junk transcripts and improving computational efficiency of cell demultiplexing. The use of "soft-clipped region of the aligned read" from minimap2 to extract Cell barcode/UMI data for demultiplexing is interesting. I would like the authors to clarify further the following:*

• Is the barcode whitelist used for cell demultiplexing the entire ~650,000 10X 5' chromium cell barcodes, or only the CellRanger / EmptyDrop filtered barcodes?

*We used CellRanger filtered barcode. We added the following sentence to the manuscript. “Accordingly, the soft-clipped portion of each read was extracted and compared to the whitelist of filtered cell barcodes identified by cellranger (Methods) from the short-read single-cell sequencing.” (Page 3)*

• Was there any other filtering done? Did the authors confirm the length of the filtered transcripts are roughly as expected of assigned exon alignments? I expect there still to be significant number of PCR artefacts that might reduce the accuracy of the counts generated.

*We removed PCR artifacts by aligning reads to genome and retaining only those reads which align to our target exons. By aligning reads to our target gene and filtering reads with exon coverage, we were able to remove most of PCR artifacts. For example, the size distribution of RACK1 amplicon of HEK293T wild-type has clear peak at its expected size. An example is shown below of the size distribution. We have noted these parameters in the Methods (Page 16, “Long read transcripts:”) and the Results (Page 4).*

*“Size of the RACK1 amplicon from HEK293T cells aggregated in the distribution with mean 1126 and 5% and 95% quantiles of sizes length population are 1044 and 1212 respectively, which is expected range.”*

• Can the following text on page 4 please be further clarified? "Using long read sequencing of target cDNAs, we characterized the transcript isoform diversity in each individual cell. Prior to this quantification step, the 10bp UMI sequence immediately following each cell barcode was reviewed. For any given set of reads that had the same barcode and UMI, these duplicate sequences were discarded." This is not a trivial task and no mention of UMI count analysis is given in the methods section.

*We have provided additional description of the UMI consolidation technique in the Methods section.*

*“Per the 10X single cell library preparation process, we know that the 10bp UMI is located directly adjacent to the 16bp cell barcode. Having established the start position of the best matching barcode as above, the start position of the UMI can be deduced. For reads sharing the same barcode their 10bp UMI is evaluated and any exact matches discarded as duplicates. We are unable to use UMI sequences whitelisted from the short-reads due to the much sparser gene expression per cell over the entire transcriptome, versus the long reads for which our genes of interest are amplified.” (Page 17)*

*Also, we added Supplementary Figure 3 which shows distribution of UMIs per cell. This graph shows only few UMIs has low edit distance which justify our UMI deduplication process.*

• With regards to direct detection of CRISPR induced indel mutations using single cell long read sequencing.* I think the authors should consider slightly altering the language for this section. Being able to accurately identify SNV from single-cell datasets using long-read sequencer is not trivial, especially without the implementation of a Cellbarcode/UMI consensus forming pipeline. Figure 2b illustrates this point, significant frameshifts are required to align transcripts towards the points of interest. If authors want to insist on this claim, further analysis might be required, and perhaps orthogonal experiments to really look into the accuracy of calling SNVs.

*Per the reviewer’s suggestion, we revised the sentence as the reviewer suggested.*

*“Beyond its application on identifying transcript isoforms, we demonstrated that this approach also enables one to directly assess the presence of CRISPR indels in the transcript. Future work will test if this method can also assess single nucleotide variants in the transcript.” (Page 12)*

• Authors should add in methods section on how exactly the mutation rates from long-read sequencing data for each gRNA target was calculated (Explain more on the method used to generate supplementary Table 7).

*We added method section with a more detailed description as shown below.*

*“CRISPR induced mutation analysis: For each gRNA, we set the analysis window as 2bp around the Cas9 cleavage site. For each aligned long read, we extracted alignment positions and aligned bases within the analysis window. Sequences which perfectly matched the reference sequence were considered wild-type and sequences with insertions, deletions or base substitutions were assigned as mutations. We performed this analysis for each gRNA for each cell and summarized the mutation rates per gRNA.” (Page 18)*

Minor comments:

The PTPRC biology text needs to be refined, how and when T-cells and NK cells express CD45 isoforms is a bit complicated. Authors should perhaps use naïve vs activated/memory T-cells CD45 isoform status as an example for the important biological implications of having the means of studying PTPRC isoforms.

*We appreciate the reviewer’s comment. We revised the paragraph to better describe the underlying relationships as the reviewer suggested. The revised text is shown below.*

*“Naïve T cells preferentially express isoforms including variable exons like RB. On the other hand, activated or memory T-cells undergo more exon skipping and express RO as a major isoform.” (Page 7)*

With regards to the comparison of transcriptome between RACK1 CRISPR edited clusters (Fig1c-d): Perhaps the authors should provide a UMAP (generated by short-read data) colour/labelled by isoform profiles C1-C9, so the viewers can visualise the global transcriptional changes are.

*We added UMAP plots based on transcript isoform cluster in Supplementary Figure 7.*

Authors might want to give 1 sentence background on the PTPRC isoform profile of Jurkat cells.

*We appreciate the reviewer’s comment. We added a sentence for Jurkat cell PTPRC isoform profile.*

*“Similar to the previous single-cell CRISPR screen result, wild-type cells demonstrated abbreviated transcripts (i.e. RO, RB) resembling the isoform profile of naïve primary T cells, while HNRNPLL RNP-treated cells expressed the longer transcript isoforms (i.e. RABC, RBC)” (Page 11)*

For the comparison of gRNA HNRNPLL vs standard Jurkat (Figures 4 & 5), I would have liked to see the scRNA-seq data (UMAPs) but labelled by isoform splice profile. Or perhaps there is no global transcriptional changes? Also, for Figure 4c, it's hard to directly compare the 4 Jurkat profiles by comparing 4 different heatmap figure, though I understand this might be given the nature of the data.

*Per the Reviewer’s suggestion, we added UMAP plots based on transcript isoform cluster in Supplementary Figure 7. As the reviewer mentions, the HNRNPLL knock-out lead to dramatic changes in the transcript isoform in PTPRC but there was less evidence of global transcriptional changes.*

With regards to Jurkat PTPRC isoform splicing experiments. Authors demonstrate the utility of their method by showing how the isoform splicing profile of PTPRC can be studied following genetic perturbations of splice factors. The splicing of CD45 is a surrogate of many key functional effector properties of T-cells, and so this application would attract the interest of the immunology community.

I believe a killer experiment would be to FACS sort PBMC T-cells into fractions of CD45RA+CDRO- , CD45RA-CD45RO+, and naïve B-cells (CD45ABC), to then be processed using authors methodology. This would act as great orthogonal validation of their isoform results (protein matching PTPRC gene profile), a result that an immunologist with limited technical expertise of single-cell methodologies will get excited about. Though I understand this would lack the CRISPR component thus acts more towards evalutating the robustness of the isoform splicing analysis pipeline.

*We appreciate the Reviewer for suggesting this experiment. For this initial proof-of-concept demonstration, we are primarily focused on showing results across applications including cellular immune regulation. As a methods manuscript, this proof-of-concept provides a broader context of applications that may interest researchers in different disciplines such as immunology. For future studies, we will report on the results of studies geared towards specific biological questions such as the Reviewer has described.*

--------------------------------------------------------------------------------------------------------------

Reviewer #2:

In this study, Kim et al assess how gene edits at splicing sites and gene knockouts of splicing factors contribute to the alternative splicing of several genes. Their experiments combine single cell library preparation with long-read (nanopore) sequencing to quantify CRISPR-modified transcript structure. The authors first present how gene edits near the splice junctions of RACK1 exons affect transcript structure, quantified via targeted nanopore cDNA sequencing. Having demonstrated initial feasibility, the authors then target splicing factors and evaluated their impact of the isoform diversity generated by PTPRC transcription.

Although their approach interesting, my enthusiasm for the work is limited due to concerns regarding the experimental quality and presentation of their work. In its current form, the data is not adequately described to enable interpretation of the experiments. In addition, several of the gene editing experiments rely on the activity of single CRISPR guide RNAs at a target site which is inadequate to make definitive comparisons.

*We appreciate the Reviewer’s feedback. We have responded to all points raised during the review.*

Major concerns:

1. Several recent studies have described issues regarding errors in single-cell barcode and UMI sequencing with nanopore (e.g. scCOLOR-seq, Philpott et al., Nature Biotechnology, 2021). The authors should use their short-read sequencing to quantify the long-read error rates and provide convincing analysis that their cosine similarity method is accurately able to call barcodes and UMIs. Some parts of the manuscript (e.g. p. 4, lines 21 - 25) hint at this type of analysis but no clear metrics are provided.

*Addressing the Reviewer’s comment, we clarify how this study did incorporate an analysis of short-read sequence data of single cell libraries and their cell barcodes. This analysis of short reads was used to identify cell barcodes with high quality sequence and then evaluate the long-read cell barcode errors. This part of the method is summarized in the manuscript on Page 3 and 4. We provide additional material in the manuscript to clarify the metrics (Supplementary Figure 2).*

*First, we created the whitelist of cell barcodes based on short-read sequencing. Next, we used the cosine similarity method to match the cell barcodes from the long sequence read with those determined from the short read white-list. For each pair, we calculated edit-distance between our reads and barcodes from whitelist and this result was used to quantify how two sequences are dissimilar to one another. We established a very stringent cut-off for barcode matching. This criteria allows only one base indel or two substitutions (edit-distance < 3) to be considered a potential match.*

*To address the Reviewer’s point, we added an edit-distance histogram comparing our long-read sequence and matching barcode (Supplementary Figure 2). This result provides metrics for possible long-read cell barcode errors. Most of cell barcodes from long-read had a perfect match (Edit-distance = 0) with barcodes from the short-read based whitelists.*

2. The authors emphasize that their method is able to capture the CRISPR-induced mutations at splice junctions but do not show indel data. This is puzzling. To me, the obvious analysis is to correlate the indels with the changes in splicing. Was this done? In particular, did some indels affect splicing more than others? Some evidence is shown in Figure 2B, but given its centrality to the scientific questions being asked, this should be done in a systematic and quantitative fashion.

*In response to this comment, we provide additional results about identifying the edit genotype versus the changes in the transcript. Current methods for single cell CRISPR assays rely on single cell RNA-Seq - verification of the altered genotype at the genomic DNA level is not done. Rather, these other methods use the guide RNA as a proxy for determining the edit for given single cell. In comparison, our single cell method does provide a direct examination and determination of an edit at single cell resolution, albeit through changes in the mRNA sequence. We have expanded this section to improve the clarity. (Page 18)*

*To summarize briefly, we targeted splicing junctions (Figure 1) and exon coding sequences (Figure 2-4) using CRISPR. For exon targeting, as CRISPR induced indels are transcribed, we were able to sequence it using cDNA (Figure 2). However, CRISPR targeting of splicing junctions lead to exons skipping. As a result, there is no detectable indel at the level of the mRNA sequence from single cells, seeing that a new alternative transcript is the direct outcome of the introduced edit.*

*Directly addressing the Reviewer’s point, we characterized the indels at the genomic DNA level for splicing junction targets. We transfected CRISPR targeting splicing junction of RACK1 exon4 or 5 to HEK293T cells. After six days, we isolated the genomic DNA of transfected cells and determined the presence of the CRISPR edit/indel present in the genomic DNA. In addition, we extracted total RNA and generated cDNA. We sequenced RACK1 amplicon from the cDNA with long-read sequencer. At the genomic DNA level, the indel was noted at a fraction of 67% for exon4 and 71% for exon5. However, the exon skipping rate as noted among the full length transcripts was significantly different, 19% for exon4 and 70% for exon5 (See the figure below, Panel A).*

*Along the lines of the Reviewer’s comment, we determined that some indels impact splicing more than others. The analysis of the indels observed from the genomic DNA showed that the mutations at exon5 sites are mostly longer deletions which removes splicing acceptors (See the figure below, Panel B, underline). Major mutations at exon4 target sites were short indels where splicing acceptors are remaining even after mutations are induced.*

*Overall, these results show how this method identifies the impact of CRISPR edits on splicing junctions with long read sequencing of cDNA from single cells. This data demonstrates the presence of the edit genotype from genomic DNA as well. In addition, as reviewer suggested some indels had a more significant impact on splicing than others.*

3. (Figure 1B) The authors do not make it clear on the specific location of guides targeting splice acceptor sites relative to the proximal splice junction. It would be helpful to know if indels were more often located within the introns or exons. Also, does the label Exon1 indicate the entire boxed region? This figure is confusing and needs improvement for clarity.

*Addressing this point, we revised the manuscript to include the guide targeting location in Supplementary Figure 1 and revised Figure 1B. As we described in our response to Point 2, we verified the CRISPR edit genotype on the DNA level as well and revised the figure accordingly to improve the clarity. Indel patterns are determined by DNA context of CRISPR target site regardless of exon or intron.*

4. (Figure 1C) It remains unclear why there are two different clusters in Figure 1C that shows a high preference for full length RACK1 transcripts, but only a single cluster in Figure 1D. The heterogeneity of transcripts that express full length RACK1 and transcripts that skip exon 5, seems to be lost. Can the authors elaborate on why this may be?

*One cluster identifies a population of cells that did not undergo the edit and as a result, these cells express a full-length transcript (Figure 1C: C2 and Figure 1D: C3). This is an expected result seeing that for any given CRISPR assay, one expects that a proportion of the cells will not demonstrate an edit. The advantage with single cell methods is that cellular variation becomes apparent. Our results also identified another cluster (C3 in Figure 1C) in which the cells have a heterozygous splicing junction edit. Therefore, alleles transcribing full length RACK1 and exon5 skipped RACK1 co-exist in a subpopulation of cells.*

5. (Figure 1) - The authors do not describe the overall quality of the single cell assays. Line 15 on page 15 does mention that 'any genes present in 3 or fewer cells were removed' which gives an idea of the lower limit of the filtering performed, however it remains unclear as to what portion of single cells met this criterion. Furthermore, for each assay, it would be helpful to know how many unique reads (as a minimum) were acquired from a single cell to support each isoform.

*Addressing this comment, we have amended the manuscript to include more details about sequencing quality data. Our approach includes both single cell short-read and long-read results. For short-read sequencing, we performed general single-cell RNA transcriptome sequencing. Cellranger and Seurat were used for cell’s gene expression analysis. We have elaborated more on the quality control metrics in the Supplementary table (Supplemantary Table 2). In average, 90% of cells passed the short-read gene expression quality control.*

*For long-read sequencing for isoform analysis, we used amplicon sequencing for various target cDNAs. The mean UMI counts per cell ranged from 7 (HNRNPLL edited Jurkat cell, PTPRC amplicon, Figure 4C) to 175.2 (Jurkat-CRISPR screen, MYL6 amplicon, Supplementary Figure 14). These metrics are shown in Supplementary Table 2. With amplicon sequencing of a single gene cDNA, we used every cell which had long-read sequence data for the analysis. We removed those cells without any long-read sequencing data for long-read analysis.*

6. (Figure 2C) - It is difficult to interpret why a single guide for each targeted exon outperform the other two guides in terms of efficiency. It would be helpful to know if the affect can be reproduced by two other guides with similar efficiency or have an explanation as to why the experiment would be limited single targeting guide.

*One of the figures was not clear in terms of the number of guides. To clarify this item, we revised the figure. The graphs in Figure 2C shows the efficiency of two guides targeting the same gene, SRSF5. The two guides target different site of SRSF5 gene and they have comparable efficiencies. We analyzed the mutation profile of both sites. At the target site for guide-1, cells which express SRSF5 guide-1 has higher mutation than cells without the guide. The target site of guide-2 produces a similar result. Therefore, both gRNAs targeting SRSF5 induced CRISPR edits successfully (Figure 2C and Supplementary Table 7). Also, Supplementary Figure 12 shows two different guides can induce similar splicing alteration.*

7. (Figure 2C) - Would it be possible to repeat this experiment with 3 different gRNAs that successfully generate indels? It might be misleading to only interpret data generated from a single cutting guide. It might also be useful for the authors to comment on any observable trends in successful guide location, relative to the splice junction.

*Our study demonstrates that gRNA’s for an overlapping target produced similar results across multiple genes. Specifically, we used two guide RNA per each splicing factor knock-out. We proved that both guide RNAs induced transcript isoform perturbation in Supplementary Figure 12. For this experiment we tried to knock-out splicing factor. So, we used guide RNA to target exons instead of splice junctions. Previous papers reported that successful guide design is determined by the DNA context whether it is close to splice junction or not (PMID: 29945655, 31723604).*

*References*

*Kim HK, Kim Y, Lee S, Min S, Bae JY, Choi JW, Park J, Jung D, Yoon S, Kim HH: SpCas9 activity prediction by DeepSpCas9, a deep learning-based model with high generalization performance. Sci Adv 2019, 5:eaax9249.*

*Chuai G, Ma H, Yan J, Chen M, Hong N, Xue D, Zhou C, Zhu C, Chen K, Duan B, et al: DeepCRISPR: optimized CRISPR guide RNA design by deep learning. Genome Biol 2018, 19:80.*

8. (Figure 2) - For the single cell RBP-knockout experiment with nanopore sequencing - it remains unclear what the criteria were for successful UMI matching. For example, what were the mean number of mismatches per UMI to reference. What were the maximum number of mismatches tolerated?

*We added more details about UMI deduplication in Methods section as noted on Page 17.*

*“Per the 10X single cell library preparation process, we know that the 10bp UMI is located directly adjacent to the 16bp cell barcode. Having established the start position of the best matching barcode as above, the start position of the UMI can be deduced. For reads sharing the same barcode their 10bp UMI is evaluated and any exact matches discarded as duplicates. We are unable to use UMI sequences whitelisted from the short-reads due to the much sparser gene expression per cell over the entire transcriptome, versus the long reads for which our genes of interest are amplified.” (Page 17)*

*As noted, the long read information had the benefits of deep sequencing through target gene-cDNA amplification. Thus, there was a substantial number of reads for a given target. We compared UMI sequence among the long-read sequences and consolidated them when they have exact match. Although short-read sequencing provides more accurate UMI sequences, we observed that the short read depth per each gene was low for any given cell. For example, in HNRNPLL edited Jurkat cell (Figure 4C), short-read sequencing had an average of 1.8 UMIs for PTPRC per cell in average. Conventional scRNA-seq and its short read data samples the entire transcriptome of a given cells. As a result, the gene expression level of PTPRC is lower than others. However, long-read sequencing had 7 UMIs per cell in average as we amplified PTPRC transcript specifically. This makes using short-read based UMI as a reference difficult.*

*In summary, short-read sequencing has full transcriptome information for gene expression analysis as a result has significantly lower reads per gene. In comparison, long-read sequencing has targeted reads for transcript and has higher reads per gene. Therefore, we couldn’t use short-read UMI as a reference. Instead, we compared UMIs in long-reads. Also, we added Supplementary Figure 3 which shows distribution of UMIs per cell. This graph shows only few UMIs has low edit distance which justify our UMI deduplication process.*

9. (Figure 3A) - It would be useful to better understand why SRSF5 and HNRNPLL knockdowns induced significant shifts in PTPRC isoform generation. Is it possible to assess the putative binding of targeted splicing factors to PTPRC transcripts using motif prediction? Can the authors elaborate on why these genes were chosen for isoform quantification?

*Pre the Reviewer’s comment, we have elaborated more on this how the splicing genes affect the PTPRC transcript. As a method paper, we chose clear example that demonstrated how one could change alternative splicing. We selected splicing factors (or RBPs) which have known effects in T-cell including PTPRC splicing regulations (citation1, 15, 16). Our results are concordant with previous reports. For example, HNRNPLL is known to bind ESS1 site at PTPRC exon4 (citation 18). We have included this information in the revised manuscript.*

*“We chose splicing factors which are expressed and play a critical role pre-mRNA processing in T cells [1, 15, 16]. For example, CELF2 and RBFOX2 regulate various pre-mRNA processing and other 14 splicing factors are known to interact with PTPRC pre-mRNA.” (Page 8)*

*“This result is concordant with previous reporting that HNRNPLL binds to ESS1 site at PTPRC exon4 [18].” (Page 10)*

*References*

*1. Gazzara MR, Mallory MJ, Roytenberg R, Lindberg JP, Jha A, Lynch KW, Barash Y: Ancient antagonism between CELF and RBFOX families tunes mRNA splicing outcomes. Genome Res 2017, 27:1360-1370.*

*15. Oberdoerffer S, Moita LF, Neems D, Freitas RP, Hacohen N, Rao A: Regulation of CD45 alternative splicing by heterogeneous ribonucleoprotein, hnRNPLL. Science 2008, 321:686-691.*

*16. ten Dam GB, Zilch CF, Wallace D, Wieringa B, Beverley PC, Poels LG, Screaton GR: Regulation of alternative splicing of CD45 by antagonistic effects of SR protein splicing factors. J Immunol 2000, 164:5287-5295.*

*18. Topp JD, Jackson J, Melton AA, Lynch KW: A cell-based screen for splicing regulators identifies hnRNP LL as a distinct signal-induced repressor of CD45 variable exon 4. RNA 2008, 14:2038-2049.*

10. (Figure 3A) - If eCLIP (or similar data) is available, it would be very useful to know if there is experimental evidence for the direct binding of the targeted RBP splicing factors to PTPRC transcripts. This information would be particularly useful in distinguishing direct interaction of splicing factors to transcripts versus indirect interactions through other RBP intermediates.

*→ We appreciate reviewer’s suggestion. The direct binding of HNRNPLL to PTPRC mRNA is previously reported (citation 18). We agree that any knock-down or knock-out experiments are not able to distinguish if target gene induces direct or indirect interaction with the transcript. However, the results from this gene knock-out screen is still meaningful as we can determine gene interaction regardless of whether the change in the transcript is direct or indirect.*

*Reference*

*18. Topp JD, Jackson J, Melton AA, Lynch KW: A cell-based screen for splicing regulators identifies hnRNP LL as a distinct signal-induced repressor of CD45 variable exon 4. RNA 2008, 14:2038-2049.*

11. (Figure 3) - Why were these 14 splicing factors were chosen to be targeted? Given the large number of splicing factors, this choice should be justified.

*To prove our new methodology, we chose splicing factors which are known to affect T cell splicing events including PTPRC. We added more explanation on this in our manuscript.*

*“We chose splicing factors which are expressed and play a critical role pre-mRNA processing in T cells [1, 15, 16]. For example, CELF2 and RBFOX2 regulate various pre-mRNA processing and other 14 splicing factors are known to interact with PTPRC pre-mRNA.” (Page 8)*

*References*

*1. Gazzara MR, Mallory MJ, Roytenberg R, Lindberg JP, Jha A, Lynch KW, Barash Y: Ancient antagonism between CELF and RBFOX families tunes mRNA splicing outcomes. Genome Res 2017, 27:1360-1370.*

*15. Oberdoerffer S, Moita LF, Neems D, Freitas RP, Hacohen N, Rao A: Regulation of CD45 alternative splicing by heterogeneous ribonucleoprotein, hnRNPLL. Science 2008, 321:686-691.*

*16. ten Dam GB, Zilch CF, Wallace D, Wieringa B, Beverley PC, Poels LG, Screaton GR: Regulation of alternative splicing of CD45 by antagonistic effects of SR protein splicing factors. J Immunol 2000, 164:5287-5295.*

Minor concerns:

1. (Figure 1B) - The gene model of RACK1 is presented in 3'-5' orientation and is confusing. This can be switched.

*We revised the figure as reviewer suggested.*

2. (Figure 1B) - The red arrows, we interpret as to be pointing to the splice acceptor sites, should be labeled clearly.

*We added Supplementary Figure 1 to clarify this item.*

3. (Figure 2B) - We observed a comparative lack of positive-stranded reads with SRSF-2. It is unclear if this is due to sampling error and should be clarified.

*That was sampling error of IGV screen shot. We revised Figure 2 to meet reviewer’s comment 6.*

4. (Supplementary Table 3) - It would be useful to plot these data as a volcano plot, so that the reader can quickly access the number of differentially expressed genes per experiment.

*We added the plot in Supplementary Figure 6.*

5. (Figure 4) - Can the authors please elaborate as to why electroporation with Cas9 RNPs were used in this experiment and not guide transduction into stably expressed Jurkat cells?

*We used lentiviral guide RNA transduction for multiplexed gene edits. As a demonstration for another CRISRP delivery method and validation of our result, we used the RNP method. Specifically, to confirm HNRNPLL gene we sought to test how well RNPs work compared to viral infection or DNA transfection. Moreover, this result demonstrates that different methods of CRISPR delivery can introduce similar effect on alternative splicing which eliminate any artifact that may be related to the delivery method used.*

**Second round of review**

**Reviewer 1**

The authors addressed my comments well and clearly, however, I still believe the authors language in the discussion of the result section for “direct single cell detection of CRISPR induced indel mutations with long read” needs to be further elaborated, specifically on the limitations/caveat of their claim.

Authors state the following: “Beyond its application on identifying transcript isoforms, we demonstrated that this approach also enables one to directly assess the presence of CRISPR indels in the transcript. Future work will test if this method can also assess single nucleotide variants in the transcript.” Figure 2b & 2c convincingly demonstrates that within an aligned window frame of target – so within a k-mer spectrum of known target and knowledge of which sample had the intended gRNA or not - you can largely evaluate the presence of CRISPR indels. This aprior knowledge of targeted analysis seemed to be essential towards putting forth the claim stated above by authors. A sentence or two should be added to relay that you can’t blindly use de novo approaches to detect CRISPR modifications from single-cell long-read sequencing, this is important to communicate as it would then suggest that perhaps this method is not ready for pooled CRISPR screens or other high-throughput CRISPR screen approaches which rely on de novo analysis (methods where you have no identifiers for intended CRISPR targets). I suspect Cellbarcode/UMI mediated consensus pipeline (contig analysis) is required for the claim put forth by authors due to the heavy nanopore read sequencing error rate.

Otherwise, if authors truly think direct single-cell detection of CRISPR mutations by nanopore-seq is truly robust enough, then further experimentation might be required. I could be convinced by an in-silico experiment where they merge the reads across all captures and then attempt to demultiplex the cells to their correct sample purely based on CRISPR genotypes. This kind of experiment is likely required to unrefutably assert their claim without language alterations.

**Reviewer 2**

All comments are addressed.

**Authors Response**

**Point-by-point responses to the reviewers’ comments:**

Reviewer #1: The authors addressed my comments well and clearly, however, I still believe the authors language in the discussion of the result section for "direct single cell detection of CRISPR induced indel mutations with long read" needs to be further elaborated, specifically on the limitations/caveat of their claim.

Authors state the following: "Beyond its application on identifying transcript isoforms, we demonstrated that this approach also enables one to directly assess the presence of CRISPR indels in the transcript. Future work will test if this method can also assess single nucleotide variants in the transcript."

Figure 2b & 2c convincingly demonstrates that within an aligned window frame of target - so within a k-mer spectrum of known target and knowledge of which sample had the intended gRNA or not - you can largely evaluate the presence of CRISPR indels. This aprior knowledge of targeted analysis seemed to be essential towards putting forth the claim stated above by authors. A sentence or two should be added to relay that you can't blindly use de novo approaches to detect CRISPR modifications from single-cell long-read sequencing, this is important to communicate as it would then suggest that perhaps this method is not ready for pooled CRISPR screens or other high-throughput CRISPR screen approaches which rely on de novo analysis (methods where you have no identifiers for intended CRISPR targets). I suspect Cellbarcode/UMI mediated consensus pipeline (contig analysis) is required for the claim put forth by authors due to the heavy nanopore read sequencing error rate. Otherwise, if authors truly think direct single-cell detection of CRISPR mutations by nanopore-seq is truly robust enough, then further experimentation might be required. I could be convinced by an in-silico experiment where they merge the reads across all captures and then attempt to demultiplex the cells to their correct sample purely based on CRISPR genotypes. This kind of experiment is likely required to unrefutably assert their claim without language alterations.

*We thank the reviewer for the positive feedback. As the reviewer suggested, we clarified that we used prior knowledge of which sample had the intended gRNA for the analysis. We added the following description in the Discussion section.*

*“The sequence of expressed guide RNA in each cell, which defines the analysis window for CRISPR indels, enables this analysis.” (Page 12)*
